# Construction of novel multi-epitope-based diagnostic biomarker HP16118P and its application in the differential diagnosis of *Mycobacterium tuberculosis* latent infection

**DOI:** 10.1186/s43556-024-00177-z

**Published:** 2024-04-29

**Authors:** Jie Wang, Fan Jiang, Peng Cheng, Zhaoyang Ye, Linsheng Li, Ling Yang, Li Zhuang, Wenping Gong

**Affiliations:** 1 Beijing Key Laboratory of New Techniques of Tuberculosis Diagnosis and Treatment, Institute of Tuberculosis Research, Senior Department of Tuberculosis, The Eighth Medical Center of PLA General Hospital, 17#Heishanhu Road, Haidian District, Beijing, 100091 China; 2 Department of Clinical Laboratory, The Eighth Medical Center of PLA General Hospital, Beijing, 100091 China; 3 Section of Health, No. 94804 Unit of the Chinese People’s Liberation Army, Shanghai, 200434 China; 4 Hebei North University, ZhangjiakouHebei, 075000 China; 5 Resident standardization training cadet corps, Air Force Hospital of Eastern Theater, Nanjing, 210002 China

**Keywords:** Tuberculosis (TB), Latent tuberculosis infection (LTBI), Multi-epitope-based diagnostic biomarker (MEBDB), Machine learning (ML), Quadratic discriminant analysis (QDA)

## Abstract

Tuberculosis (TB) is an infectious disease that significantly threatens human health. However, the differential diagnosis of latent tuberculosis infection (LTBI) and active tuberculosis (ATB) remains a challenge for clinicians in early detection and preventive intervention. In this study, we developed a novel biomarker named HP16118P, utilizing 16 helper T lymphocyte (HTL) epitopes, 11 cytotoxic T lymphocyte (CTL) epitopes, and 8 B cell epitopes identified from 15 antigens associated with LTBI-RD using the IEDB database. We analyzed the physicochemical properties, spatial structure, and immunological characteristics of HP16118P using various tools, which indicated that it is a hydrophilic and relatively stable alkaline protein. Furthermore, HP16118P exhibited good antigenicity and immunogenicity, while being non-toxic and non-allergenic, with the potential to induce immune responses. We observed that HP16118P can stimulate the production of high levels of IFN-γ^+^ T lymphocytes in individuals with ATB, LTBI, and health controls. IL-5 induced by HP16118P demonstrated potential in distinguishing LTBI individuals and ATB patients (*p*=0.0372, AUC=0.8214, 95% CI [0.5843 to 1.000]) with a sensitivity of 100% and specificity of 71.43%. Furthermore, we incorporated the GM-CSF, IL-23, IL-5, and MCP-3 induced by HP16118P into 15 machine learning algorithms to construct a model. It was found that the Quadratic discriminant analysis model exhibited the best diagnostic performance for discriminating between LTBI and ATB, with a sensitivity of 1.00, specificity of 0.86, and accuracy of 0.93. In summary, HP16118P has demonstrated strong antigenicity and immunogenicity, with the induction of GM-CSF, IL-23, IL-5, and MCP-3, suggesting their potential for the differential diagnosis of LTBI and ATB.

**Supplementary Information:**

The online version contains supplementary material available at 10.1186/s43556-024-00177-z.

## Introduction

Tuberculosis (TB) is a chronic infectious disease caused by *Mycobacterium tuberculosis* (MTB), with pulmonary TB being the most common form. According to the World Health Organization (WHO) Global Tuberculosis Report 2023, there were 10.6 million new TB cases and 1.3 million TB-related deaths worldwide in 2022 [1]. By 2022, 30 high-burden countries will account for 87% of the world's tuberculosis cases, with China ranking third with 7.1%, after India (27%) and Indonesia (10%) [2]. Previous studies have shown that about one-third of the global population infected with TB develops active tuberculosis (ATB), while the remaining 90% develop latent tuberculosis infection (LTBI) [3]. LTBI refers to a special state in which individuals infected with MTB do not exhibit clinical manifestations or radiographic changes of active TB but test positive for a tuberculin skin test (TST) [4]. Without timely diagnosis and intervention, individuals with LTBI have a 5-10% lifetime risk of progressing to ATB. However, when individuals with LTBI are coinfected with human immunodeficiency virus (HIV), the risk can be as high as 10%, significantly higher than in HIV-negative populations [5–7]. Epidemiological investigations have shown that 85-90% of newly diagnosed ATB cases are attributable to LTBI [4]. Therefore, early detection and differential diagnosis of LTBI form the foundation for preventing and controlling the transmission of TB.

Currently, the detection methods for LTBI include TST and interferon-γ release assays (IGRAs) [8]. The traditional TST uses the purified protein derivative tuberculin (PPD) as the antigen, which results in high false-positive rates among individuals vaccinated with Bacillus Calmette-Guérin (BCG) and cannot distinguish between LTBI and ATB patients [9]. In recent years, new TST diagnostic methods, such as Diaskintest, C-Tb Skin Test, and EC-test, have been developed using antigens like early secreted antigen target protein 6 (ESAT-6) and culture filtrate protein 10 (CFP-10) instead of traditional PPD [10]. In addition, there are five IGRA test kits, including T-SPOT.TB, QFT-GIT, QFT-Plus, LIAISONQFT-Plus, and LIOFeron TB/LTBI [7, 11, 12]. These IGRAs and the new TST diagnostic methods use CFP-10 and ESAT-6 as stimulating antigens, significantly improving the diagnostic sensitivity and specificity for MTB infection, but still cannot distinguish between LTBI and ATB patients. Therefore, identifying effective LTBI diagnostic candidates and their application to the differential diagnosis of LTBI are essential for improving the sensitivity and specificity of LTBI diagnosis, reducing the probability of developing active TB, and promoting TB prevention and control.

Research has shown that antigens from the region of difference (RD) and latency-associated antigens of MTB hold the most potential as target antigens for distinguishing LTBI from ATB [7]. In the preliminary study, we screened 21 candidate antigens (LTBI-RD-related antigens) that belong to both the RD-related antigens and latent infection stage antigens, including Rv1511, Rv1736c, Rv1737c, Rv1978, Rv1980c, Rv1981c, Rv2031c, Rv2626c, Rv2653c, Rv2654c, Rv2656c, Rv2657c, Rv2658c, Rv2659c, Rv2660c, Rv3425, Rv3429, Rv3872, Rv3873, Rv3878, and Rv3879c [13–31]. We further studied the Th1-type helper T lymphocyte (HTL) epitopes, cytotoxic T lymphocyte (CTL) epitopes, and the number of interferon-gamma (IFN-γ) ^+^ T lymphocytes in the peptide pool induced by these candidate antigens in mice with ATB, LTBI, and healthy controls (HCs). The results showed that ATB mice had five Th1-dominant peptides, seven CTL-dominant peptides, and four peptides pool-induced IFN-γ^+^ T lymphocyte frequencies higher than those in LTBI and HC mice [32]. Additionally, we successfully constructed multi-epitope vaccines (MEVs) and multi-epitope-based diagnostic biomarkers (MEBDBs) based on the above antigens, demonstrating their good immunogenicity in LTBI, ATB, and HC populations [33–38]. Therefore, immunodominant epitopes of LTBI-RD-related antigens have potential applications in diagnosing and preventing TB.

In this study, we predicted and screened potential immunodominant HTL, CTL, and B cell epitopes based on 15 LTBI-RD-related antigens. We connected these epitopes using linkers and adjuvants to construct an MEBDB. The physicochemical properties, immunological characteristics, and spatial structures of MEBDB were analyzed using bioinformatics and immunoinformatics techniques, and the immune responses of MEBDB were simulated. MEBDB was expressed and purified *in vitro*. The immunological characteristics of MEBDB were validated using enzyme-linked immunospot assays (ELISPOT) and high-throughput liquid protein analysis, and its diagnostic performance was evaluated in three groups: LTBI, ATB, and HCs. The MEBDB constructed in this study provides new candidate diagnostic molecules for the differential diagnosis of LTBI.

## Results

### Prediction of dominant HTL, CTL, and B cell epitopes and construction of the diagnostic molecule HP16118P

**Fig. 1:**
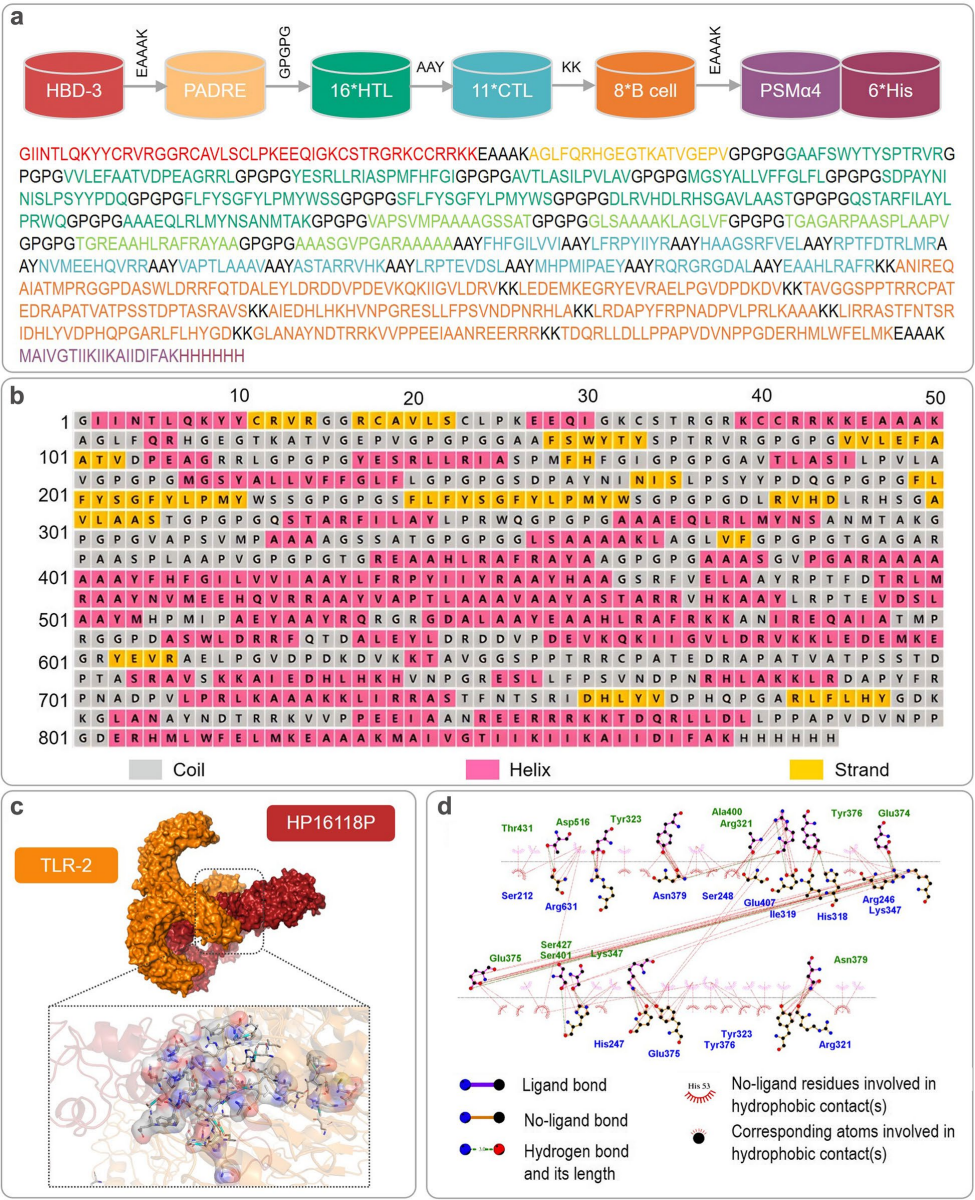
Construction and secondary and tertiary structure analysis of the HP16118P. **a** Schematic diagram of HP16118P construction. The molecule contains 844 amino acids, with green representing HTL epitopes, blue representing CTL epitopes, and orange representing B cell epitopes. PSMα4 is a TLR-2 agonist, and PADRE and HBD-3 are auxiliary peptides. **b** Secondary structure of HP16118P. Pink highlights indicate α-helices, yellow regions represent β-sheets, and gray regions represent coils. **c** Visualization of the molecular docking between HP16118P and TLR-2 using PyMOL software. **d** Predicted two-dimensional representation of the interacting amino acid sites between HP16118P and TLR-2, with HP16118P represented in blue and TLR2 in green. The interacting amino acid sites between HP16118P and TLR2 may provide potential mechanisms for its immunomodulatory effects.

Based on previous research, we further selected 15 LTBI-RD related antigens with potential for distinguishing LTBI (Table S1), including Rv1511 [39], Rv1736c [13], Rv1737c [14, 15], Rv1978 [28], Rv1980c [21], Rv1981c [22], Rv2031c [29–31], Rv2626c [16–19], Rv2656c [40], Rv2659c [20], Rv3425 [25–27], Rv3429 [28], Rv3873 [22], Rv3878 [23], and Rv3879c [24]. Based on the selected 15 antigens, we further predicted and selected 16 dominant HTL epitopes, 11 dominant CTL epitopes, and eight dominant B cell epitopes (Table 1), constituting the central part of MEBDB. To enhance the immune effect and targeting of MEBDB, we added epitope adjuvant peptides human beta-defensin-3 (HBD-3) and PADRE at the amino terminus, Toll-like receptor 2 (TLR-2) agonist phenol-soluble modulin α 4 (PSMα4) at the carboxyl terminus, and 6 His tags (HHHHHH) to connect all epitopes, forming a novel MEBDB candidate named HP16118P (Fig. 1a).

**Table 1:** List of information on the dominant epitopes of HTL, CTL and B cells constituting HP16118P

Antigen	Peptide	Allele	Start	End	Length	Percentile rank ^a^	Antigenicity scores ^b^	IFN-γ scores ^c^	Immunogenicity score^d^	Allergenicity
HTL peptide										
Rv1736c	GAAFSWYTYSPTRVR	HLA-DQA1*02:01/DQB1*05:02	106	120	15	0.14	0.9205	0.4379	NA	Non
	VVLEFAATVDPEAGRRL	HLA-DQA1*05:01/DQB1*02:01	365	381	17	0.49	1.3268	0.3351	NA	Non
	YESRLLRIASPMFHFGI	HLA-DRB1*14:04	456	472	17	0.2	0.8424	0.1577	NA	Non
Rv1737c	AVTLASILPVLAV	HLA-DQA1*06:01/DQB1*03:03	76	88	13	0.34	0.8199	0.3890	NA	Non
	MGSYALLVFFGLFL	HLA-DRB1*15:02	94	107	14	0.36	0.7974	0.1674	NA	Non
Rv1980c	SDPAYNINISLPSYYPDQ	HLA-DRB3*02:02	46	63	18	0.41	1.0470	2	NA	Non
Rv1981c	FLFYSGFYLPMYWSS	HLA-DRB1*15:02	163	177	15	0.08	0.8181	1	NA	Non
	SFLFYSGFYLPMYWS	HLA-DRB1*15:02	162	176	15	0.08	0.8237	1	NA	Non
Rv2659c	DLRVHDLRHSGAVLAAST	HLA-DQA1*05:01/DQB1*03:01	312	329	18	0.44	0.9292	2	NA	Non
Rv3429	QSTARFILAYLPRWQ	HLA-DQA1*02:01/DQB1*05:02	153	167	15	0.28	0.7639	0.1360	NA	Non
	AAAEQLRLMYNSANMTAK	HLA-DRB3*02:02	25	42	18	0.41	0.7112	0.5479	NA	Non
Rv3873	VAPSVMPAAAAGSSAT	HLA-DQA1*03:01/DQB1*06:01	304	319	16	0.31	1.0173	0.2373	NA	Non
Rv3878	GLSAAAAKLAGLVF	HLA-DQA1*03:01/DQB1*06:01	11	24	14	0.43	0.7126	0.6727	NA	Non
	TGAGARPAASPLAAPV	HLA-DQA1*05:01/DQB1*03:01	252	267	16	0.09	0.7544	0.6624	NA	Non
Rv3879	TGREAAHLRAFRAYAA	HLA-DQA1*02:01/DQB1*05:02	670	685	16	0.01	0.7194	0.9824	NA	Non
	AAASGVPGARAAAAA	HLA-DQA1*05:01/DQB1*03:01	365	379	15	0.09	0.9853	0.7878	NA	Non
CTL peptide										
Rv1736c	FHFGILVVI	HLA-C*06:02	468	476	9	0.32	1.0655	NA	0.2502	Non
	LFRPYIIYR	HLA-A*30:01	629	637	9	0.3	0.9571	NA	0.2374	Non
	HAAGSRFVEL	HLA-C*03:04	210	219	10	0.23	0.9214	NA	0.1396	Non
Rv2031c	RPTFDTRLMR	HLA-A*11:01	32	41	10	0.21	1.3628	NA	0.1265	Non
Rv2626c	NVMEEHQVRR	HLA-A*33:03	92	101	10	0.11	0.8767	NA	0.1287	Non
Rv2656c	VAPTLAAAV	HLA-C*01:02	47	55	9	0.03	0.7845	NA	0.1173	Non
Rv2659c	ASTARRVHK	HLA-A*30:01	168	176	9	0.06	0.8908	NA	0.2048	Non
Rv1511	LRPTEVDSL	HLA-C*07:02	281	289	9	0.13	1.2094	NA	0.0938	Non
Rv3429	MHPMIPAEY	HLA-C*07:02	1	9	9	0.04	0.8170	NA	0.0303	Non
Rv3879c	RQRGRGDAL	HLA-B*15:01	479	487	9	0.23	1.0645	NA	0.1747	Non
	EAAHLRAFR	HLA-A*33:03	673	681	9	0.02	1.0320	NA	0.1845	Non
B-cell peptide										
Rv1737c	LRDAPYFRPNADPVLPRLKAAA		182	203	22	NA	NA	NA	NA	Non
Rv1978	ANIREQAIATMPRGGPDASWLDRRFQTDALEYLDRDDVPDEVKQKIIGVLDRV		4	56	53	NA	NA	NA	NA	Non
Rv2031c	LEDEMKEGRYEVRAELPGVDPDKDV		42	66	25	NA	NA	NA	NA	Non
Rv2656c	TAVGGSPPTRRCPATEDRAPATVATPSSTDPTASRAVS		2	39	38	NA	NA	NA	NA	Non
Rv2659c	AIEDHLHKHVNPGRESLLFPSVNDPNRHLA		264	293	30	NA	NA	NA	NA	Non
Rv1511	LIRRASTFNTSRIDHLYVDPHQPGARLFLHYGD		31	63	33	NA	NA	NA	NA	Non
Rv3425	GLANAYNDTRRKVVPPEEIAANREERRR		90	117	28	NA	NA	NA	NA	Non
Rv3879c	TDQRLLDLLPPAPVDVNPPGDERHMLWFELMK		632	663	32	NA	NA	NA	NA	Non

^a^Percentile rank ranking of selected epitopes: select epitopes with a rank score of <0.5

^b^Antigenicity score: select epitopes with an antigenicity score of >0.7

^c^IFN-γ score: select epitopes with a positive score

^d^Immunogenicity score: select epitopes with an immunogenicity score >0

*NA* Not available

### Prediction of the physicochemical properties and immunological characteristics of HP16118P

The physicochemical properties of the HP16118P molecule are crucial for its immunological functions. We used the Expasy ProtParam server to predict the physicochemical properties of HP16118P. The results showed that HP16118P has a molecular weight of 90265.44 Da, a theoretical isoelectric point of 9.84, a GRAVY index of 75.09, an instability index of 43.02, and an overall average hydrophilicity of -2.7 (Table S2). The in vivo half-life showed that HP16118P has a half-life of over 10 hours in *E. coli*. Using the Protein-Sol server, the predicted isoelectric point of HP16118P was 10.23, with a solubility of 0.382. In summary, HP16118P is a stable and hydrophilic protein with moderate solubility.

Furthermore, the HP16118P molecule used for LTBI discrimination diagnosis must possess good antigenicity and immunogenicity to induce effective immune responses and should not be allergenic or toxic. Immunogenicity analysis of HP16118P revealed an immunogenicity score of 6.43254 and antigenicity scores of 0.7381 and 0.60063 (Table S2). This indicates that HP16118P has good immunogenicity and can induce immune responses in immune cells. Additionally, both methods predicted that HP16118P is non-allergenic, and results from the Toxin Pred server indicated that HP16118P is non-toxic. In conclusion, HP16118P is a non-toxic, non-allergenic protein with good immunogenicity and antigenicity.

### Prediction of the spatial structure of HP16118P and the interactions between HP16118P and TLR-2, and simulation of the HP16118P-induced immune response

We used PSIPRED to predict the secondary structure of HP16118P (Fig. 1b) and found that the HP16118P molecule contains 844 amino acids, with 41% α-helices, 7% β-sheets, and 50% random coils. We further employed four structure prediction servers (Rebetta, Swiss model, AlphaFold2, and I-TASSER) to predict the tertiary structure of HP16118P. Subsequently, we obtained five potential tertiary structure models and performed structural optimization using GalaxyWEB. Each model underwent quality assessment using ERRAT, VERIFY 3D, PROCHECK, and WHATCHECK methods (Table 2). Our results revealed that prior to GalaxyWEB optimization, model 5 predicted by I-TASSER exhibited the best quality: (1) ERRAT provided a quality score of 79.1209 and passed the VERIFY 3D test (at least 80% of the amino acids have scored >= 0.1 in the 3D/1D profile); (2) PROCHECK identified 844 residues, with evaluations including 9 items, comprising 7 Errors, 2 Warnings, and 0 Passes; (3) The Ramachandran plot displayed percentages of 65.10% in the core region (favored region), 27.60% in the allowed region, 4.50% in the generously allowed region, and 2.80% in the disallowed region; (4) WHATCHECK results comprised 48 items, of which 10 were Errors, 20 were Warnings, and 18 were Passes. Interestingly, after GalaxyWEB optimization, we found that model 4 exhibited the best quality: (1) ERRAT provided a quality score of 73.8538 and passed the VERIFY 3D test (at least 80% of the amino acids have scored >= 0.1 in the 3D/1D profile); (2) PROCHECK identified 844 residues, with evaluations including 9 items, comprising 4 Errors, 3 Warnings, and 1 Pass; (3) The Ramachandran plot displayed percentages of 81.50% in the core region, 13.40% in the allowed region, 2.20% in the generously allowed region, and 2.80% in the disallowed region; (4) WHATCHECK results comprised 46 items, of which 4 were Errors, 15 were Warnings, and 27 were Passes.

**Table 2:** Prediction of HP16118P spatial structural features using different models and algorithms

	**Assessment methodology**	**Level 1 indicators**	**Level 2 indicators**	**Rebetta**	**Swiss model**	**AlphaFold2**	**I-TASSER**	**GalaxyWEB**
Model 1	ERRAT			Errat Failed. No results.	80.2469	39.1691	77.0202	71.8362
	VERIFY 3D	At least 80% of the amino acids have scored >= 0.1 in the 3D/1D profile.		Fail	Fail	Fail	Fail	Fail
	PROCHECK	Ramachandran plot	core	100.00%	84.40%	41.20%	68.70%	80.60%
			allow	0.00%	13.00%	18.70%	24.80%	14.20%
			gener	0.00%	2.60%	13.30%	4.20%	2.10%
			disall	0.00%	0.00%	26.90%	2.40%	3.10%
		residues		7	89	844	844	844
		evaluations	Error	0	5	7	7	4
			Warning	0	1	1	2	3
			Pass	8	2	1	0	1
	WHATCHECK	Whole		41	47	47	48	46
		Error		4	8	8	10	5
		Warning		4	15	17	19	16
		Pass		33	24	22	19	25
Model 2	ERRAT			Errat Failed. No results.	68.8889	51.567	79.0382	75.5611
	VERIFY 3D	At least 80% of the amino acids have scored >= 0.1 in the 3D/1D profile.		Fail	Fail	Fail	Fail	Fail
	PROCHECK	Ramachandran plot	core	100.00%	87.30%	55.20%	65.10%	81.80%
			allow	0.00%	12.70%	19.90%	28.20%	13.40%
			gener	0.00%	0.00%	10.70%	5.10%	1.60%
			disall	0.00%	0.00%	14.20%	1.60%	3.10%
		residues		7	64	844	844	844
		evaluations	Error	0	3	7	7	4
			Warning	0	2	1	2	3
			Pass	8	3	1	0	1
	WHATCHECK	Whole		41	47	47	49	46
		Error		4	6	8	11	4
		Warning		4	12	17	18	15
		Pass		33	29	22	20	27
Model 3	ERRAT			too small	68.5714	59.5982	68.2741	75.5556
	VERIFY 3D	At least 80% of the amino acids have scored >= 0.1 in the 3D/1D profile.		too small	Fail	Fail	Fail	Fail
	PROCHECK	Ramachandran plot	core	too small	91.00%	56.60%	49.10%	81.20%
			allow	too small	6.40%	25.20%	36.40%	13.60%
			gener	too small	1.30%	13.10%	7.50%	2.50%
			disall	too small	1.30%	5.10%	7.00%	2.70%
		residues		too small	91	844	844	844
		evaluations	Error	too small	4	6	6	4
			Warning	too small	1	2	3	3
			Pass	too small	3	1	0	1
	WHATCHECK	Whole		too small	47	47	48	46
		Error		too small	6	8	11	4
		Warning		too small	14	16	19	15
		Pass		too small	27	23	18	27
Model 4	ERRAT			too small	53.8462	59.3596	65.7895	73.8538
	VERIFY 3D	At least 80% of the amino acids have scored >= 0.1 in the 3D/1D profile.		too small	Fail	Fail	Pass	Pass
	PROCHECK	Ramachandran plot	core	too small	71.40%	56.30%	46.40%	81.50%
			allow	too small	21.40%	26.60%	38.50%	13.40%
			gener	too small	7.10%	12.20%	9.70%	2.20%
			disall	too small	0.00%	4.90%	5.40%	2.80%
		residues		too small	35	844	844	844
		evaluations	Error	too small	4	6	7	4
			Warning	too small	2	1	2	3
			Pass	too small	2	1	0	1
	WHATCHECK	Whole		too small	44	47	49	46
		Error		too small	7	8	11	4
		Warning		too small	7	15	19	15
		Pass		too small	30	24	19	27
Model 5	ERRAT			too small	89.3617	75.3463	79.1209	72.5248
	VERIFY 3D	At least 80% of the amino acids have scored >= 0.1 in the 3D/1D profile.		too small	Fail	Fail	Pass	Fail
	PROCHECK	Ramachandran plot	core	too small	84.40%	57.50%	65.10%	81.20%
			allow	too small	15.60%	26.40%	27.60%	13.60%
			gener	too small	0.00%	12.10%	4.50%	2.10%
			disall	too small	0.00%	4.00%	2.80%	3.10%
		residues		too small	55	844	844	844
		evaluations	Error	too small	3	6	7	4
			Warning	too small	3	1	2	3
			Pass	too small	2	1	0	1
	WHATCHECK	Whole		too small	46	47	48	46
		Error		too small	7	8	10	5
		Warning		too small	14	15	20	15
		Pass		too small	25	24	18	26

**Fig. 2:**
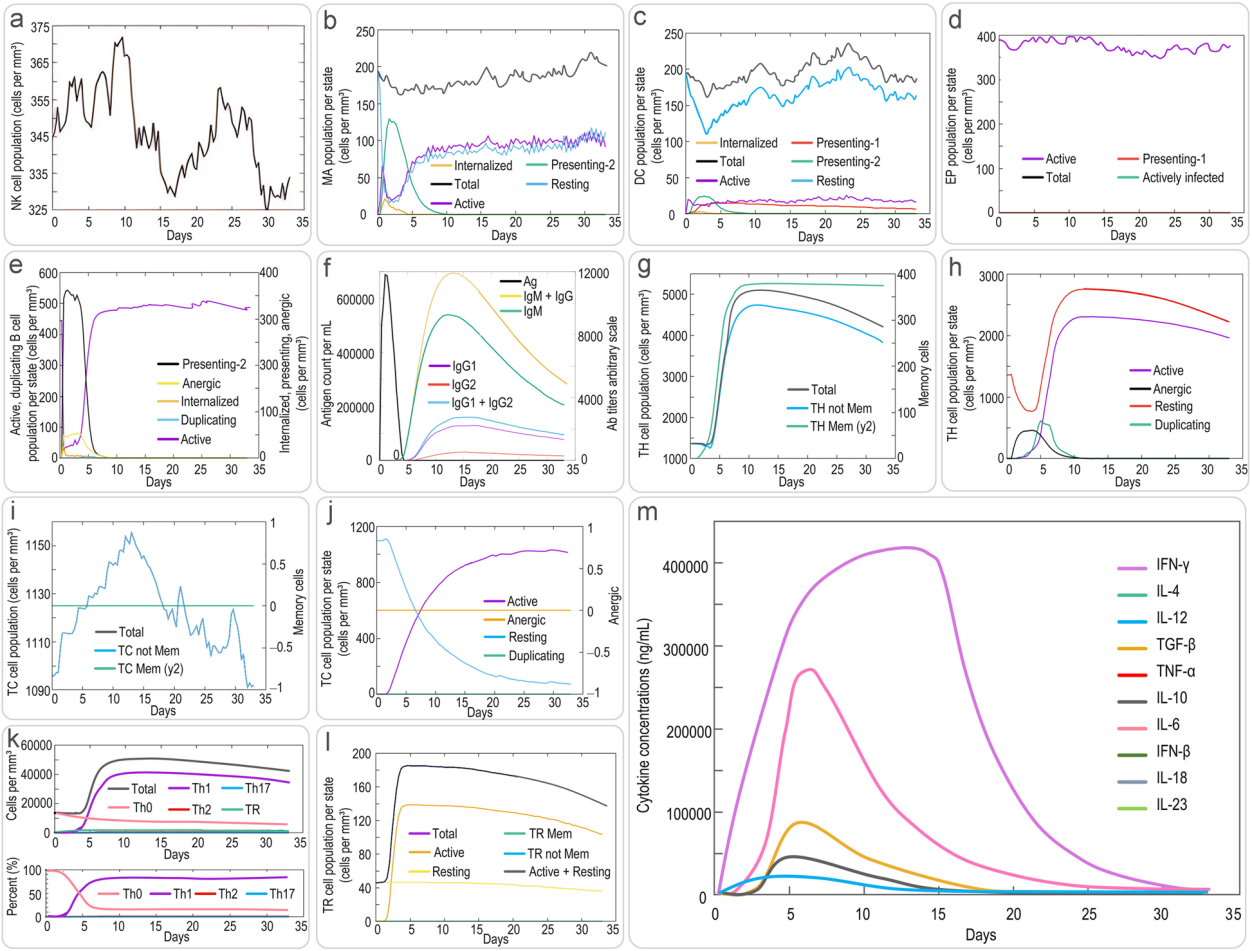
Prediction of innate and adaptive immune responses induced by HP16118P using the C-ImmSim server. The C-ImmSim server was used to predict the innate natural killer cells (**a**), macrophages (**b**), dendritic cells (**c**), epithelial cells (**d**), B cells (**e**), antibody levels (**f**), memory Th cells (**g**, classified by memory cell count), effector Th cells (**h**, classified by active, resting, non-responsive, and replicative counts), memory TC cells (**i**, classified by memory cell count), effector TC cells (**j**, classified by active, resting, non-responsive, and replicative counts), Th cell subtypes (**k**, including Th0, Th1, Th2, Th17), TR subgroups (**l**), and cytokine levels (**m**) induced by HP16118P after immune stimulation in humans. Abbreviations: TH Mem, memory T helper cells; TC, cytotoxic T cell; NK cells, natural killer cells; MA, macrophage; DC, dendritic cell; EP, epithelium; TR, regulatory T cells; Mem, Memory; TGF-β, transforming growth factor-β

Considering the TLR-2 targeting ability of the designed HP16118P in this study, we analyzed the amino acid sites involved in the interaction between HP16118P and TLR-2 using the ClusPro 2.0 online server. LigPlot^+^ visualization results showed that HP16118P and TLR-2 could dock closely and interact with each other, with a center energy of -1066.9 kcal/mol and a Lowest Energy of -1436 kcal/mol (Fig. 1c). Further analysis revealed 12 pairs of interacting amino acid residues (Fig. 1d). Subsequently, we used the C-ImmSim server to simulate the immune response induced by HP16118P. We found that HP16118P successfully stimulated the immune system, demonstrating the ability to influence the production of specific antibodies and various cytokines by immune cells. The results showed that (1) HP16118P can activate natural NKs, maintaining their numbers between 325-375 cells/mm^3^ (Fig. 2a). HP16118P also stimulated the proliferation and differentiation of macrophages (MA) and DCs, inducing the proliferation peak of presenting-2 type MA cells (Fig. 2b) and DCs (Fig. 2c). Unlike DCs, the number of resting and active MA cells stabilized at approximately 90 cells/mm^3^ on the eighth day after HP16118P-induced immune simulation (Fig. 2b). HP16118P also significantly activated epithelial cells (EPs) (Fig. 2d). Furthermore, HP16118P stimulated the differentiation and proliferation of B lymphocytes, rapidly increasing the number of presenting-2 type B cells, with the peak of active B lymphocytes reaching on the fifth day after stimulation (Fig. 2e). Interestingly, we also observed a significantly high level of HP16118P-specific IgG and IgM antibodies produced by HP16118P-induced active B lymphocytes (Fig. 2f).

In addition, we analyzed the immune effects of HP16118P on specific immune cells. The results showed that the peak number of memory helper T lymphocytes (Th) induced by HP16118P can reach 4500 cells/mm^3^ (Fig. 2g), while the number of active Th cells reached its peak on the tenth day after immune stimulation (Fig. 2h). In contrast to Th cells, the ability of HP16118P to induce the production of memory cytotoxic T lymphocytes (Tc) by the human immune system remained stable after immune activation (Fig. 2i). The number of active TC cells peaked on the fifteenth day after immune stimulation, while the number of resting TC cells showed an opposite trend (Fig. 2j). Excitingly, we found that HP16118P can induce the differentiation of T lymphocytes into Th1-type lymphocytes, mediating a strong Th1-type immune response (Fig. 2k). Moreover, we observed that regulatory T cells (Tregs/TR) rapidly increased and peaked on the second day after immune stimulation by HP16118P (Fig. 2l). Finally, we analyzed the ability of HP16118P to induce immune cells to produce cytokines. We found that HP16118P can generate high levels of IFN-γ, transforming growth factor-β (TGF-β), interleukin 11 (IL-12), and IL-2 in human immune cells (Fig. 2m).

### Successful in vitro expression of HP16118P and increased number of IFN-γ+ T lymphocytes in HCs, ATB, and LTBI individuals

**Fig. 3:**
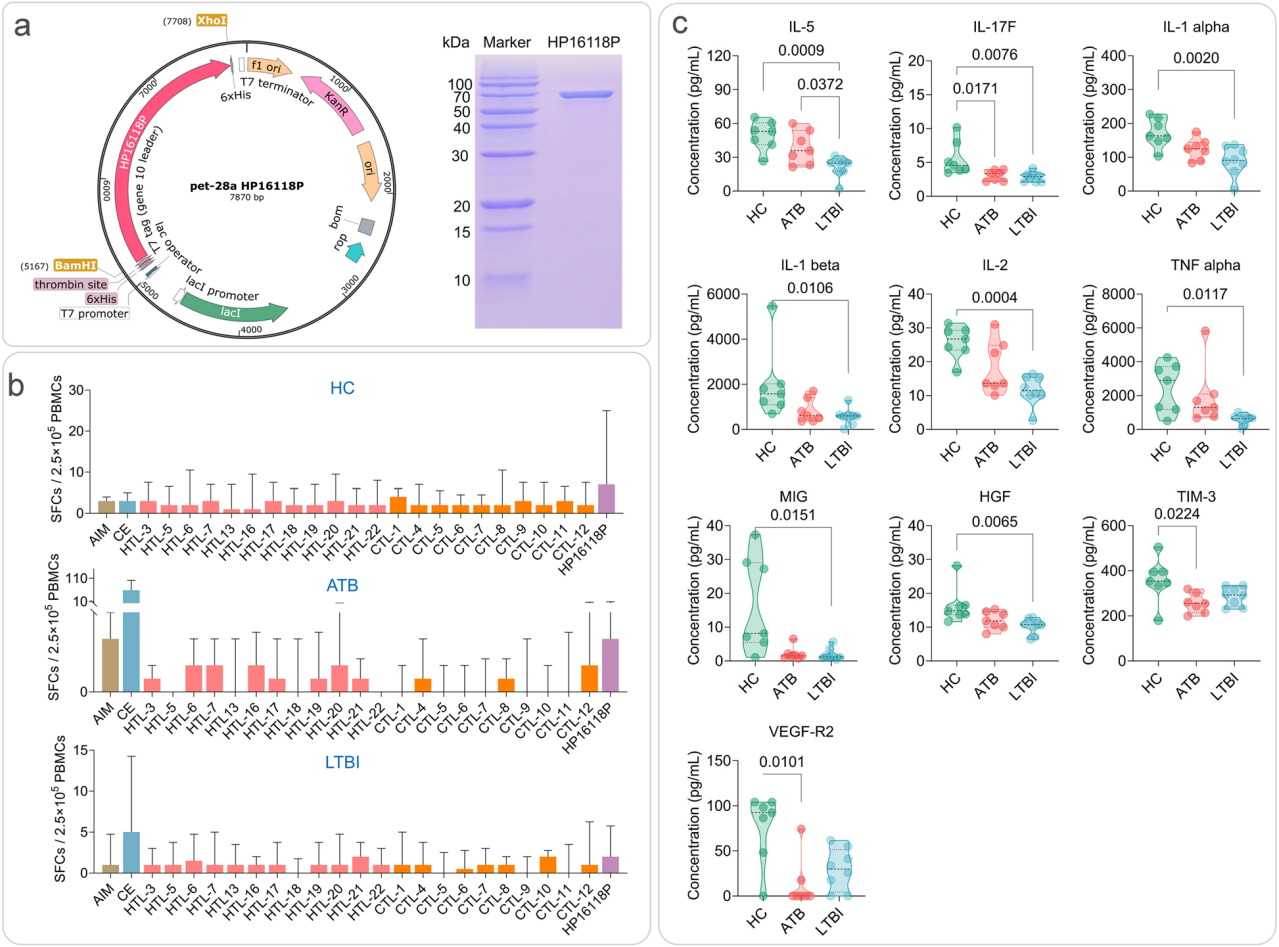
Construction and expression of HP16118P and the number of IFN-γ^+^ T lymphocytes and cytokines induced by HP16118P in HC, ATB, and LTBI groups. **a** Schematic diagram of the recombinant plasmid of HP16118P and protein purification gel electrophoresis. **b** Detection of IFN-γ^+^ T lymphocytes by enzyme-linked immunospot assay (ELISPOT) after HP16118P stimulation of PBMCs. HP16118P, 12 HTL epitope peptides, 10 CTL epitope peptides, AIM medium (negative control), and CE (positive control) were used to stimulate PBMCs from healthy individuals (*n* = 23), ATB patients (*n* = 19), and LTBI individuals (*n* = 24). The frequency of IFN-γ^+^ T lymphocytes was detected using the ELISPOT method. **c** Differential cytokine induction by HP16118P in HC, ATB, and LTBI groups. PBMCs from HCs (*n*=7), ATB (*n*=8), and LTBI (*n*=7) individuals were stimulated with HP16118P *in vitro*, and the culture supernatant was collected after 48 hours for high-throughput liquid chromatography protein analysis to detect the expression levels of 35 cytokines. Results showed significant differences in cytokines, including IL-5, IL-17F, IL-1α, IL-1β, IL-2, TNF-α, MIG, HGF, TIM-3, VEGF-R2, among the three groups. The data were analyzed using the non-parametric Kruskal-Wallis test, with a significance level of *P* < 0.05. The data are presented as medians and interquartile ranges. AIM, auto-induction medium; SFCs, spot-forming cells; HTL, helper T lymphocytes; CTL, cytotoxic T lymphocytes; CE, the fusion protein of CFP-10 and ESAT-6; ATB, active tuberculosis; LTBI, latent tuberculosis infection; PBMC, peripheral blood mononuclear cells

We inserted the HP16118P gene sequence between the BamH I and Xho 1 restriction sites of the pET28a(+) plasmid while keeping the other gene sequences of the pET-28a(+) vector unchanged to construct the recombinant plasmid pET-28a(+)-HP16118P (Fig. 3a). Polyacrylamide gel electrophoresis results showed that after three rounds of Ni column affinity chromatography, we successfully purified the fusion protein HP16118P with a molecular weight between 70-100kDa, as expected (Fig. 3a). To minimize the impact of endotoxins on the immunogenicity and biological functionality of the PP16118P protein, we employed the Beyotime Protein Endotoxin Removal Kit to eliminate endotoxins, followed by the detection of endotoxin levels using the Beyotime Chromogenic LAL Endotoxin Assay Kit. The results demonstrated that the endotoxin concentration in the purified PP16118P protein, after the removal process, was found to be below 1×10^-4^ EU/µg.

We conducted ELISPOT experiments to detect the number of IFN-γ^+^ T lymphocytes induced by HP16118P and individual HTL and CTL epitopes in every 2.5×10^5^ peripheral blood mononuclear cells (PBMCs). This experiment included 23 HCs, 24 LTBI, and 19 ATB subjects, and the number of IFN-γ^+^ T lymphocytes was measured in each group. The results (Fig. 3b) showed that, compared to the auto induction medium (AIM)-negative control stimulus, the number of IFN-γ^+^ T lymphocytes induced by HP16118P increased, but the difference was not significant (*P*>0.05). Compared to HP16118P, the number of IFN-γ^+^ T lymphocytes induced by individual HTL and CTL epitopes generally remained low. These data suggest that HP16118P can cause the proliferation of IFN-γ^+^ T lymphocytes compared to individual HTL and CTL epitopes.

### HP16118P induces high levels of cytokine secretion in PBMCs

To evaluate the consistency of the HP16118P diagnostic molecule in the computer simulation and *in vitro*-induced immune response, we performed cytokine detection on PBMCs collected from HCs, LTBI individuals, and ATB patients. Initially, HP16118P induced the secretion of 35 cytokines in PBMCs, with concentrations greater than 10000 pg/ml for tissue inhibitor of metalloprotease-1 (TIMP-1), more significant than 1000 pg/ml for granulocyte-macrophage colony-stimulating factor (GM-CSF), IL-6, IL-8, monocyte chemoattractant protein-1 (MCP-1), macrophage inflammatory protein 1β (MIP-1β), and tumor necrosis factor α (TNF-α), and greater than 100 pg/ml for IL-1α, IL-10, IL-23, T cell immunoglobulin and mucin domain-containing protein 3 (TIM-3), and vascular endothelial growth factor A (VEGF-A) (Fig. S1). These data indicate that HP16118P possesses strong immunogenicity and can induce various cytokine production in immune cells.

Further analysis of the differences in HP16118P-induced cytokines among the three groups revealed that IL-5 (*P*=0.0009), IL-17F (*P*=0.0076), IL-1α (*P*=0.0020), IL-1β (*P*=0.0106), IL-2 (*P*=0.0004), TNF-α (*P*=0.0117), monokine induced by gamma (MIG) (*P*=0.0151), and hepatocyte growth factor (HGF) (*P*=0.0065) were significantly lower in the LTBI group compared to the HC group. IL-17F (*P*=0.0171), TIM-3 (*P*=0.0224), and vascular endothelial growth factor receptor 2 (VEGF-R2) (*P*=0.0101) induced by HP16118P were significantly lower in the ATB group compared to the HC group (Fig. 3c). IL-5 (*P*=0.0372) induced by HP16118P was substantially lower in the LTBI group compared to the ATB group (Fig. 3c). Furthermore, we compared the differences in the levels of 35 cytokines produced by PBMCs from the ATB, LTBI, and HC groups in response to PBS and HP16118P stimulation using the R package "autoReg". Our results revealed that compared to the negative control PBS, HP16118P significantly induced higher levels of G-CSF, GM-CSF, IFN-γ, IL-1α, IL-1β, IL-10, IL-12p70, IL-17F, IL-2, IL-21, IL-22, IL-23, IL-31, IL-4, IL-5, IL-6, IP-10, MCP-1, MCP-3, MIP-1β, PD-1, TNF-α, and VEGF-A in PBMCs from individuals with ATB, LTBI, and/or HC (Table 3).

**Table 3:** Thirty-five cytokines levels in three populations under two interventions with PBS and HP16118P

Cytokines	Data presentation	ATB (*n*=14)			HC (*n*=14)			LTBI (*n*=16)		
		PBS (*n*=7)	HP16118P (*n*=7)	*P* value	PBS (*n*=7)	HP16118P (*n*=7)	*P* value	PBS (*n*=8)	HP16118P (*n*=8)	*P* value
G-CSF	Median (IQR)	17.80 (13.50 to 27.10)	331.45 (326.96 to 494.05)	**<.001**	17.09 ± 3.72	420.48 ± 172.73	**<.001**	33.96 ± 49.60	322.90 ± 177.94	**0.002**
GM-CSF	Median (IQR)	0.00 (0.00 to 5.83)	1460.48 (1078.05 to 2275.91)	**0.002**	0.00 (0.00 to 0.00)	1215.75 (911.34 to 1306.40)	**0.001**	0.00 (0.00 to 28.43)	1065.72 (701.82 to 1204.53)	**0.004**
HGF	Mean ± SD	13.00 ± 3.71	12.01 ± 2.67	0.577	14.29 (13.63 to 14.84)	14.84 (13.79 to 16.32)	0.7	9.64 (8.43 to 10.25)	10.75 (8.70 to 11.80)	0.093
IFN-α	Median (IQR)	0.00 (0.00 to 0.00)	0.11 (0.00 to 0.31)	0.234	0.09 (0.01 to 0.33)	0.24 (0.04 to 0.54)	0.698	0.00 (0.00 to 0.26)	0.00 (0.00 to 0.17)	0.904
IFN-γ	Median (IQR)	0.87 (0.39 to 2.38)	5.92 (2.90 to 29.51)	**0.012**	7.42 (5.35 to 8.52)	33.18 (16.19 to 60.74)	0.055	4.39 (4.39 to 4.78)	4.00 (3.20 to 18.59)	0.559
IL-1α	Mean ± SD	5.50 ± 6.16	124.00 ± 31.84	**<.001**	3.36 ± 1.05	172.72 ± 43.21	**<.001**	3.13 (2.45 to 11.39)	90.68 (69.50 to 124.84)	**0.002**
IL-1β	Median (IQR)	12.41 (7.38 to 33.05)	602.01 (437.81 to 1107.10)	**<.001**	14.71 (8.50 to 22.74)	1579.41 (1165.08 to 1916.29)	**<.001**	12.71 (4.99 to 43.80)	594.82 (391.36 to 629.30)	**0.002**
IL-10	Median (IQR)	2.90 (1.39 to 5.49)	188.39 (109.16 to 289.03)	**<.001**	2.35 (1.54 to 2.45)	186.71 (59.47 to 262.88)	**0.001**	1.47 (1.06 to 4.37)	103.94 (68.25 to 244.36)	**0.002**
IL-12p70	Median (IQR)	0.36 (0.34 to 0.38)	0.96 (0.69 to 1.52)	**<.001**	0.45 (0.38 to 0.49)	2.85 (1.22 to 16.02)	**0.002**	0.41 (0.33 to 0.45)	0.68 (0.42 to 3.29)	0.126
IL-13	Mean ± SD	2.20 ± 0.54	5.19 ± 3.42	0.061	2.72 ± 0.33	7.32 ± 3.54	**0.014**	2.39 ± 0.16	3.55 ± 1.03	**0.015**
IL-17F	Mean ± SD	2.13 ± 0.80	3.05 ± 0.78	**0.048**	2.94 ± 1.21	5.58 ± 2.53	**0.028**	2.05 ± 0.83	2.84 ± 0.72	0.062
IL-2	Mean ± SD	4.27 ± 2.26	18.39 ± 7.80	**0.002**	5.70 ± 1.78	25.85 ± 4.85	**<.001**	4.76 (4.42 to 5.42)	11.48 (10.05 to 15.29)	0.082
IL-21	Mean ± SD	8.82 ± 5.63	18.48 ± 4.96	**0.005**	17.02 ± 5.34	24.66 ± 7.52	**0.049**	8.02 ± 8.03	18.05 ± 6.02	**0.013**
IL-22	Median (IQR)	9.46 (9.33 to 10.18)	13.35 (11.12 to 15.99)	**0.011**	16.21 ± 3.41	35.13 ± 23.14	0.074	11.55 ± 4.08	13.27 ± 2.47	0.326
IL-23	Mean ± SD	6.98 ± 3.78	332.38 ± 198.71	**0.005**	10.77 (10.39 to 11.88)	370.42 (245.98 to 436.41)	**0.005**	10.12 (8.32 to 11.10)	228.48 (133.24 to 295.07)	**0.01**
IL-3	Mean ± SD	9.38 ± 1.31	10.35 ± 1.43	0.206	10.89 ± 1.12	11.92 ± 1.38	0.152	9.93 ± 1.50	10.77 ± 1.34	0.257
IL-31	Mean ± SD	1.91 ± 0.97	3.84 ± 1.02	**0.003**	3.55 ± 1.41	4.99 ± 1.30	0.071	3.29 ± 1.50	4.13 ± 1.31	0.253
IL-4	Mean ± SD	2.94 ± 1.28	11.45 ± 5.33	**0.005**	6.72 (5.81 to 7.06)	14.79 (14.15 to 17.64)	**0.004**	4.47 ± 2.22	8.11 ± 4.41	0.056
IL-5	Mean ± SD	3.62 ± 2.93	38.60 ± 14.85	**<.001**	2.35 ± 1.37	49.42 ± 13.05	**<.001**	1.70 (0.00 to 3.25)	24.80 (17.75 to 26.36)	**0.004**
IL-6	Mean ± SD	3521.82 ± 2806.94	8106.69 ± 2618.21	**0.008**	1870.48 ± 1663.45	15640.37 ± 8202.15	**0.004**	2832.08 ± 3729.50	9198.03 ± 6471.14	**0.03**
IL-8	Mean ± SD	4047.66 ± 1415.14	4207.92 ± 1428.47	0.837	6437.56 ± 886.20	5181.07 ± 2818.90	0.297	6169.91 (5366.49 to 6578.14)	6241.64 (3480.94 to 6726.94)	0.798
IL-9	Mean ± SD	32.84 ± 7.44	31.19 ± 6.40	0.665	38.61 (35.85 to 40.58)	36.79 (31.13 to 38.27)	0.337	31.21 ± 7.75	28.81 ± 5.64	0.49
IP-10	Median (IQR)	3.08 (2.75 to 3.39)	3.99 (3.52 to 4.63)	0.053	5.85 ± 2.84	27.15 ± 22.16	**0.044**	3.02 (2.57 to 4.15)	3.16 (2.60 to 8.77)	0.721
MCP-1	Median (IQR)	895.93 (736.03 to 2934.16)	1212.04 (389.52 to 2133.46)	0.535	4704.88 ± 3117.15	1633.29 ± 2025.52	**0.049**	1317.71 (427.45 to 3620.39)	1261.64 (508.38 to 1975.10)	0.721
MCP-3	Mean ± SD	106.77 ± 55.58	52.29 ± 11.90	**0.041**	109.06 ± 79.77	48.00 ± 31.24	0.097	71.36 ± 44.13	36.20 ± 18.80	0.067
MIG	Median (IQR)	1.26 (0.98 to 1.49)	1.56 (1.21 to 1.95)	0.318	4.50 ± 3.33	16.52 ± 14.29	0.069	1.20 (0.92 to 1.40)	1.22 (0.92 to 2.62)	0.721
MIP-1α	Mean ± SD	83.92 ± 25.68	71.24 ± 22.66	0.347	157.35 ± 55.52	84.32 ± 44.02	**0.018**	89.88 ± 41.51	91.59 ± 48.27	0.94
MIP-1β	Mean ± SD	1449.22 ± 700.81	4799.87 ± 1483.02	**<.001**	2150.89 (1941.74 to 2924.59)	7945.38 (4251.00 to 10742.42)	0.053	1265.81 ± 886.36	4912.32 ± 3114.64	**0.013**
PD-1	Mean ± SD	2.85 ± 1.47	12.05 ± 6.53	**0.009**	5.37 ± 2.35	14.85 ± 6.10	**0.005**	2.63 ± 1.62	8.52 ± 4.61	**0.008**
SDF-1α	Median (IQR)	23.71 (8.41 to 59.72)	51.34 (32.51 to 59.82)	0.607	25.01 ± 15.54	47.05 ± 40.70	0.219	83.54 ± 44.75	102.78 ± 54.31	0.452
TIM-3	Mean ± SD	282.51 ± 64.62	256.02 ± 43.57	0.386	350.65 ± 69.92	358.47 ± 98.13	0.866	242.40 ± 85.93	286.76 ± 41.90	0.21
TIMP-1	Mean ± SD	54972.72 ± 21062.35	34651.69 ± 13061.73	0.051	89259.60 (65187.51 to 136669.11)	56257.84 (39887.43 to 69856.31)	0.073	71822.68 (43546.99 to 76033.65)	39979.36 (27456.73 to 48734.38)	0.161
TNF-α	Median (IQR)	57.93 (23.71 to 72.26)	1299.13 (947.03 to 1893.37)	**<.001**	31.92 ± 10.71	2483.02 ± 1463.95	**0.004**	47.01 ± 70.63	590.75 ± 305.89	**0.001**
VEGF-A	Mean ± SD	841.61 ± 364.54	131.33 ± 38.63	**0.002**	1172.88 ± 405.70	167.79 ± 75.58	**<.001**	1179.86 ± 392.71	306.30 ± 365.52	**<.001**
VEGF-R2	Mean ± SD	36.79 ± 36.54	13.12 ± 27.76	0.197	80.46 (51.38 to 89.36)	92.43 (67.35 to 101.15)	0.521	49.51 ± 39.77	29.38 ± 23.09	0.236

Bold indicates *P* value <0.05

### Correlation analysis of HP16118P-induced cytokines

We performed principal component analysis (PCA) and correlation analysis to understand further the potential relationship between HP16118P-induced cytokines in healthy individuals, ATB patients, and LTBI individuals. The results showed that in LTBI individuals (Fig. S2a), the cumulative variation percentages of the concentrations of 35 cytokines induced by HP16118P on principal component 1 (PC1) and PC2 were 57.45% and 19.12%, respectively. We observed positive correlations between IL-1α, IL-1β, IL-5, IL-13, IL-21, IL-23, programmed cell death-1 (PD-1), granulocyte-colony stimulating factor (G-CSF), TNF-α, GM-CSF, and IFN-α, while IL-10 and IFN-γ tended to cluster together and showed negative correlations with MIP-1β and IL-6. Interestingly, we also found negative correlations between VEGF-A and most other cytokines. In ATB patients (Fig. S2b), the cumulative variation percentages of the concentrations of 35 cytokines induced by HP16118P on PC1 and PC2 were 47.25% and 20.29%, respectively. We found that IL-10, IFN-γ, GM-CSF, G-CSF, and IL-12p70 clustered together. In contrast, IL-1α, IL-1β, IL-2, IL-17F, and IL-22 clustered together, showing positive correlations among cytokines within each cluster and negative correlations between IL-21 and most other cytokines. In HCs (Fig. S2c), the cumulative variation percentages of the concentrations of 35 cytokines induced by HP16118P on PC1 and PC2 were 44.56% and 26.13%, respectively. We also found that IL-17F and IL-1β clustered together. In contrast, IL-13, IL-21, and GM-CSF clustered together, showing positive correlations among cytokines within each cluster and negative correlations between VEGF-A and most other cytokines.

### Discriminatory diagnostic performance analysis of HP16118P in HC, ATB, and LTBI populations

**Fig. 4:**
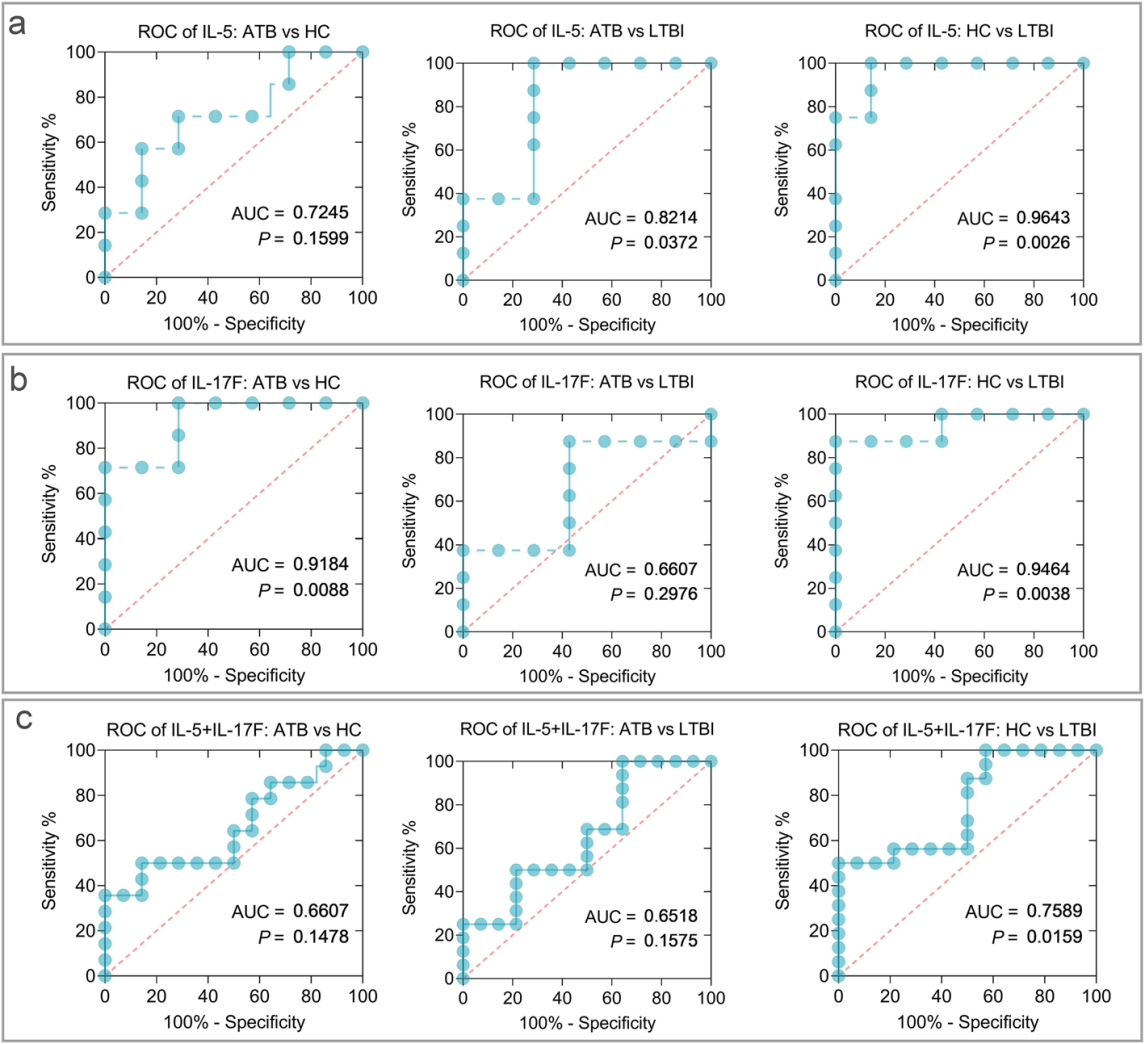
ROC curves of HP16118P-induced IL-5 and IL-17F cytokines for differential diagnosis of ATB, LTBI, and HCs groups. ROC curves were used to determine the sensitivity and specificity of HP16118P-induced cytokines IL-5 (**a**), IL-17F (**b**), and their combination (**c**) in the differentiation of ATB and LTBI using the Wilson/Brown method. Each graph indicates the AUC and *P*-value, with *P* < 0.05 indicating a significant difference

Based on the aforementioned results, we further selected the differentially significant IL-5 and IL-17F as biomarkers for discriminating diagnosis among ATB, LTBI, and HCs. The results are shown in Table S3: (1) Induced IL-5 by HP16118P was able to distinguish LTBI individuals from ATB (*P*=0.0372, area under the curve (AUC) =0.8214, 95% CI [0.5843 to 1.000]) and HC (*P*=0.0026, AUC=0.9643, 95% CI [0.8770 to 1.000]) individuals, with sensitivity and specificity of 100% and 71.43% (ATB vs. LTBI) and 100% and 85.71% (HC vs. LTBI), respectively (Fig. 4a). (2) Induced IL-17F by HP16118P was able to distinguish ATB individuals from HC (*P*=0.0088, AUC=0.9184, 95% CI [0.7716 to 1.000]) individuals, with sensitivity and specificity of 71.43% and 85.71% (Fig. 4b). IL-17F could also distinguish LTBI individuals from HC (*P*=0.0038, AUC=0.9464, 95% CI [0.8299 to 1.000]) individuals, with sensitivity and specificity of 87.50% and 85.71%, respectively. (3) The combination of IL-5 and IL-17F was able to distinguish LTBI individuals from HC (*P*=0.0159, AUC=0.7589, 95% CI [0.5842 to 0.9336]) individuals, with sensitivity and specificity of 50.00% and 85.71% (Fig. 4c).

### ATB and LTBI differential diagnostic model based on 15 machine learning algorithms and HP16118P-induced cytokines

**Fig. 5:**
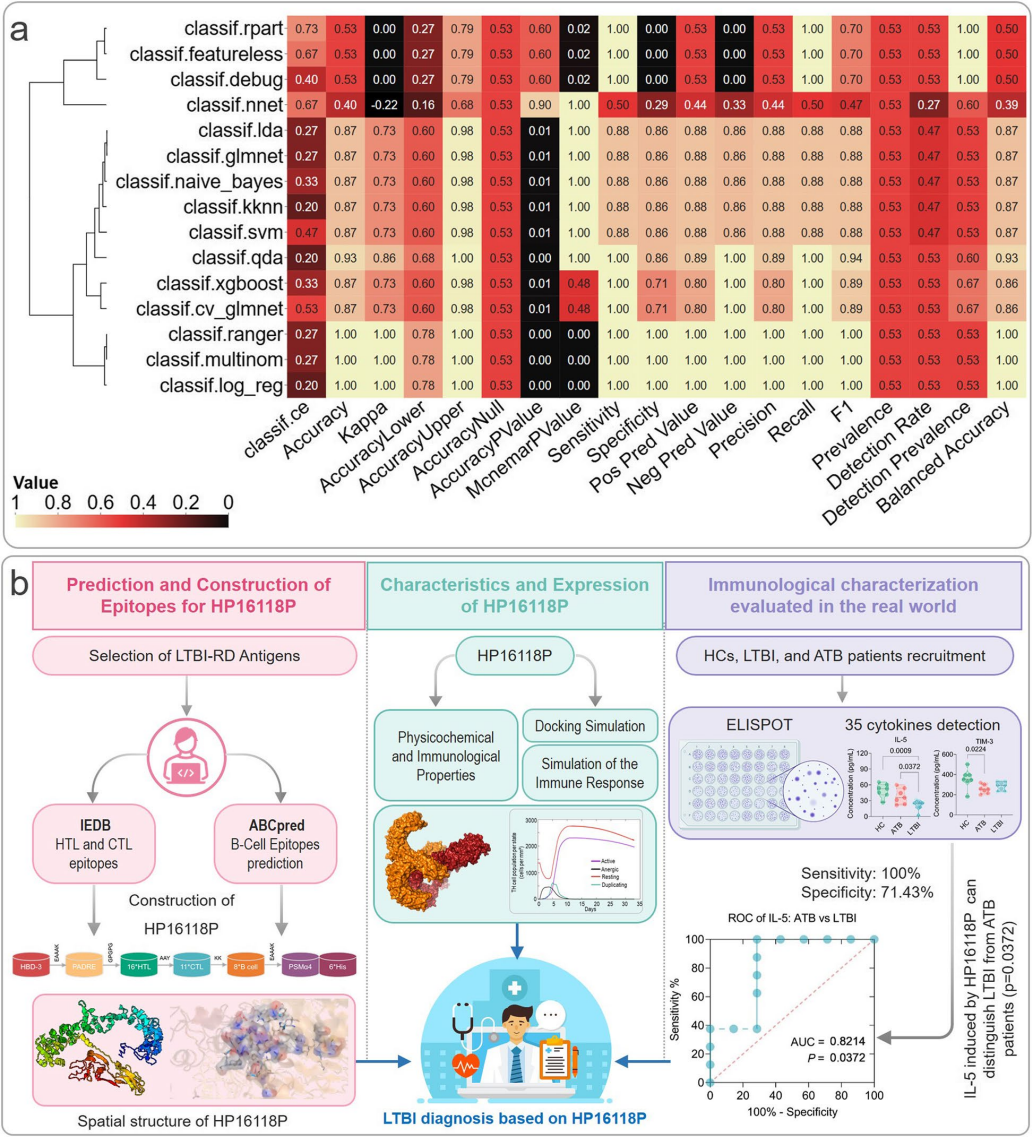
Performance of 15 Machine Learning Models and Development of HP16118P Biomarker. **a** Heatmap. From four selected cytokines, a heatmap displays the performance metrics of 15 machine learning models. The QDA model is emphasized for its superior diagnostic capabilities. Key terms: Classif.ce reflects multiclass classification; Kappa denotes Cohen’s coefficient; Accuracy Lower/Upper are confidence intervals; Accuracy Null is the baseline accuracy; Accuracy *P* Value assesses statistical significance; McNemar *P* Value compares model performance; Recall, or sensitivity, measures correct positive predictions; and F1 score combines precision and recall values. **b** Experimental Flowchart: The creation of the multi-epitope biomarker HP16118P advances tuberculosis diagnosis by differentiating ATB from LTBI. The discovery of IL-5 as a specific differentiating cytokine highlights the biomarker’s utility and its potential to enhance immune response, showcasing a significant breakthrough in tuberculosis management and global health impact

Using the R package “autoReg”, we conducted univariate, multivariate, and stepwise logistic regression analyses on the expression levels of 35 cytokines induced by HP16118P in the ATB and LTBI groups to select potential models for distinguishing LTBI and ATB. The results (Table S4) demonstrated that the cytokines GM-CSF (*P* = 0.999, OR = 1.16, 95%CI [0.00-5.5918E+140]), IL-23 (*P* = 0.999, OR = 0.27, 95%CI [0.00-Inf]), IL-5 (*P* = 0.999, OR = 0.00 95%CI [0.00-Inf]), and MCP-3 (*P* = 0.999, OR = 0.01 95%CI [0.00-Inf]) were included in the stepwise logistic regression model. Subsequently, these four cytokines were integrated into the construction of the machine learning models, and the detailed results of 19 diagnostic performance indicators for 15 machine learning models were presented in Table S5. The heatmap of the data results was shown in Fig. 5a. The Quadratic Discriminant Analysis (QDA) model was selected as the optimal model due to its excellent diagnostic performance (Classif. ce = 0.2000, Accuracy = 0.9333, Kappa = 0.8649, Accuracy Lower = 0.6805, Accuracy Upper = 0.9983, Accuracy Null = 0.5333, Accuracy *P* Value = 0.0011, McNamara *P* Value = 1.0000, Sensitivity = 1.0000, Specificity = 0.8571, Positive Predictive Value = 0.8899, Negative Predictive Value = 1.0000, Precision = 0.8899, Recall = 1.0000, F1 = 0.9412, Prevalence = 0.5333, Detection Rate = 0.5333, Detection Prevalence = 0.6000, and Balanced Accuracy = 0.9268).

## Discussion

The discrimination and diagnosis of LTBI have always been challenging in the early detection and precise prevention of TB. Compared to the early PPD test, the newly developed TST methods and IGRAs have significantly improved the sensitivity and specificity of diagnosis by replacing PPD with ESAT-6 and CFP-10, thus excluding interference from BCG vaccination and environmental non-tuberculous mycobacterial infections [41–44]. Unfortunately, these methods cannot distinguish between latent and active TB, resulting in the inability to differentiate between ATB and LTBI.

Our efforts to identify biomarkers for the discrimination of LTBI and ATB led us to select 15 promising antigens from a previous identification of 21 LTBI-RD-related antigens Fig. 5b. These selected antigens allowed us to construct our novel LTBI diagnostic biomarkers by identifying dominant epitopes associated with HTL, CTL, and B cells [34, 45, 46]. Given epitopes’ inherent limitations in immunogenicity, we enhanced our biomarker with the TLR-2 agonist PSMα4 and helper epitopes HBD-3 and PADRE, augmenting their immunogenic potential and stability [47–51]. These adjuncts not only improve immune response but also signify advances in MTB control and vaccine strategies [47, 52–54].

Advances in bioinformatics and immunoinformatics have revolutionized the development of diagnostic biomarkers and vaccines [47, 55, 56]. Through reverse genetics, we analyzed HP16118P, an LTBI and ATB diagnostic biomarker, and found it to be stable, hydrophilic, and moderate in solubility, weighing 90265.44 Da (Fig. 5b). It exhibits strong antigenicity and immunogenicity, capable of eliciting an immune response without causing sensitization or toxicity. Simulations via the C-ImmSim server confirmed that HP16118P activates innate immune cells like NK cells, DCs, and MAs, essential for the initial defense against MTB and adaptive immune response orchestration [47, 49, 57]. Furthermore, HP16118P effectively stimulates effector and memory T lymphocytes, as well as Th1 CD4^+^ T cells, which play a vital role in MTB clearance [58]. This is supported by cytokine induction including IFN-γ, IL-6, and TGF-β, as confirmed by in vitro experiments and consistent with previous research on immune molecular markers in MTB response [59]. Our findings also align with biomarkers like IP-10, IFN-γ, IL-1ra, CCL3, VEGF, TNF-α, MCP-1, and GM-CSF relevant in TB diagnosis [60–63], indicating that HP16118P may effectively contribute to TB diagnostic approaches.

Our research extended to examining the response of 35 cytokines to HP16118P in ATB, LTBI, and HC groups. We observed significantly lower cytokine levels, namely IL-1α, IL-1β, IL-17F, IL-2, IL-5, MIG, HGF, and TNF-α, in LTBI compared to HCs, while IL-17F and TIM-3 levels were markedly reduced in ATB versus HCs. Notably, IL-5 levels were significantly reduced in the LTBI group compared to ATB, highlighting its potential as a diagnostic marker. We identified IL-5 and IL-17F as key cytokines demonstrating differential expression and representing distinct pro-inflammatory cytokine types [64], with varying levels across MTB infection stages. IL-5 distinguished LTBI from ATB with high sensitivity and specificity (100% and 71.43%, respectively), whereas IL-17F modestly differentiated ATB from HCs (71.43% sensitivity and 85.71% specificity). Previous research in Nairobi reported sensitivity and specificity rates for IL-5 and IL-17A in differentiating ATB from LTBI at 75.0%/91.7% and 66.7%/92.9%, correspondingly [65]. Our findings are supportive of the use of IL-5 as a differentiator between LTBI and ATB, although with varying results for IL-17A and IL-17F when compared to the Kenyan study.

The IL-5 cytokine response to HP16118P stimulation offers insights for differentiating LTBI from ATB. IL-5, associated with Th2 immunity and involved in eosinophil activation and B cell function [66], may display varying levels between LTBI and ATB due to distinct immune reactions. LTBI is marked by a Th1-dominated profile with lower IL-5, while ATB may exhibit increased IL-5 due to a mixed Th1/Th2 response [47, 49, 67]. Assessing IL-5 levels relative to other cytokines in response to HP16118P can help identify the stage of *Mycobacterium tuberculosis* infection. Nonetheless, IL-5 should be analyzed alongside a comprehensive cytokine profile for an accurate diagnosis [68, 69]. Further research is necessary to fully understand IL-5’s diagnostic role in TB infection. Furthermore, the IL-17 cytokine family, key players in chronic inflammation and associated diseases, is predominantly produced by Th cells [70]. Among its six members (IL-17A-F) [71], IL-17A and IL-17F were thought to act similarly due to shared receptors. However, distinct roles in mucosal immunity and allergic reactions have been observed in knockout mice studies, differentiating their biological functions [72]. This difference might explain the disparity between our findings on IL-17F and the Kenya study on IL-17A in discerning ATB from LTBI.

As machine learning (ML) becomes integral in diagnosing TB, its use in differentiating LTBI from ATB remains limited [69, 73]. Our study aimed to address this gap by comparing traditional ROC methods with ML in diagnosing latent infections. Through logistic regression, we pinpointed four cytokines (GM-CSF, IL-23, IL-5, MCP-3), with the QDA model demonstrating excellent diagnostic accuracy at 0.93. This suggests a move towards computational analyses for data potential maximization in future research. However, it's vital to consider the sample size, maintaining a minimum of ten times the number of variables to avoid overfitting—a challenge we encountered with our preliminary HP16118P validation. Our study's 19 diagnostic indicators provide a comprehensive comparison framework for future LTBI and ATB differentiation models, highlighting the need to extend beyond traditional measures like AUC, sensitivity, and specificity.

This study also has several limitations: (1) The HTL, CTL, and B cell epitopes comprising the diagnostic molecule HP16118P were not individually validated for their immunogenicity *in vitro*, but were instead based on bioinformatics and immunoinformatics analysis; (2) The sample size for evaluating HP16118P's discriminatory diagnosis of LTBI and ATB was relatively small, and further improvements are needed to enhance the stability of the ROC results; (3) Despite using 15 machine learning algorithms to construct the LTBI discriminatory diagnostic model based on the analysis of HP16118P-induced levels of 35 cytokines in different populations (ATB, LTBI, HCs), the results of multiple machine learning algorithms were missing due to the small sample size. Despite these limitations, the LTBI discriminatory diagnostic candidate HP16118P, constructed based on bioinformatics and immunoinformatics, demonstrated good discriminatory diagnostic capability in the current small sample size cohort. Its diagnostic efficacy needs further confirmation in larger sample-size studies.

## Conclusion

The biomarker HP16118P developed in this study exhibits strong antigenicity and immunogenicity for distinguishing between ATB and LTBI. It is non-allergenic and non-toxic, effectively stimulating the immune system and promoting the proliferation of B lymphocytes and T lymphocytes, producing high levels of antibodies and cytokines. The immunogenicity of HP16118P was confirmed through ELISPOT and high-throughput liquid-phase protein analysis, which demonstrated its ability to induce the production of IFN-γ^+^ T lymphocytes and various inflammatory cytokines. Additionally, the cytokine IL-5 induced by HP16118P shows potential in differentiating between LTBI and ATB individuals, thus serving as a promising candidate target for ATB and LTBI discrimination diagnosis.

## Materials and methods

### Selection of LTBI-RD antigens

The antigens comprising HP16118P were chosen based on their documented association with latent tuberculosis infection (LTBI) and their capacity to evoke an immune response in individuals with LTBI. This study selected 15 antigens with the potential for differential diagnosis from the previously screened LTBI-RD related antigens [7], including Rv1511, Rv1736c, Rv1737c, Rv1978, Rv1980c, Rv1981c, Rv2031c, Rv2626c, Rv2656c, Rv2659c, Rv3425, Rv3429, Rv3873, Rv3878, and Rv3879c. These antigens have been identified through comprehensive literature reviews, experimental evidence, and bioinformatics analysis, ensuring their relevance to TB pathogenesis and diagnosis. The amino acid sequences of these 15 proteins were downloaded in FASTA format from the National Centre for Biotechnological Information (NCBI).

### Prediction and selection of HTL epitopes

Allele Frequency Net Database was used to screen China-specific MHC-II restrictive alleles. The Immune Epitope Database (IEDB) was employed to predict dominant HTL epitopes for the Chinese population [74]. According to the literature reported, for MHC-II allelic restricted HTL epitopes, epitopes with lower percentile ranking scores have higher binding affinity to MHC-II [75]. Epitopes with percentile ranking score <0.5 or IC50 value <500nM were selected for further analysis as candidate epitopes. VaxiJen2.0 was used to predict the antigenicity of HTL epitopes [76], and epitopes with antigenicity scores>0.7 were selected. IFN epitope server was used to predict HTL epitopes with good IFN-γ inducible capability [77]. Aller TOP2.0 and AllergenFP1.0 were then used to predict the non-allergenicity of HTL epitopes with positive IFN-γ inducible capability [78, 79]. The selected epitopes from these criteria were regarded as candidate epitopes.

### Prediction and selection of CTL Epitopes

The IEDB database was used to predict dominant CTL epitopes for the Chinese population. Epitopes with a Percentile Rank score less than 0.5 were selected as candidate epitopes [75]. The immune characteristics of epitopes were predicted using the IEDB database, and epitopes with scores greater than 0 were selected as candidate epitopes [80]. VaxiJen 2.0 was used to predict the antigenicity of CTL epitopes, with a threshold set at 0.5. Epitopes with antigen scores greater than 0.7 were selected. Aller TOP2.0 and AllergenFP1.0 were further used to predict the non-allergenicity of the target mentioned above epitopes, resulting in a list of candidate epitopes for epitope molecule construction.

### Prediction and selection of B-cell epitopes

In addition to T-cell-mediated direct or cytokine-mediated indirect interactions with MTB during anti-TB infection, B-cell-mediated humoral immunity also plays an important role [81]. Therefore, the prediction and selection of B-cell epitopes were carried out simultaneously. ABCpred prediction server was used to predict linear B-cell epitopes [82]. The B-cell epitopes were sorted by score, and the higher the score, the higher the likelihood of being an epitope. To further improve the prediction accuracy of B-cell epitopes, IEDB B Cell Epitope Prediction was used. Subsequently, the B-cell epitopes predicted by different servers were compared, and the B-cell epitopes predicted by all servers were selected as the final selected epitopes.

### Construction of MEBDB

The construction of the MEBDB involved carefully selecting adjuvant and linker sequences to enhance its immune effect and targeting ability. We employed a systematic approach based on the analysis of predicted epitopes and available literature to achieve this. From the predicted epitopes mentioned above, we selected 16-18 HTL epitopes, 10-12 CTL epitopes, and 6-8 B-cell epitopes for inclusion in the MEBDB. We chose specific linker sequences for each cell type to ensure proper spatial orientation and interaction of the epitopes within the construct. GPGPG was selected as the linker for HTL epitopes, AAY for CTL epitopes, and KK for B-cell epitopes. In addition to the linkers, we incorporated adjuvants and auxiliary peptides to enhance the immunogenicity and immune response induction of HP16118P. Adjuvant PSMα4 [83], adjuvant linker EAAAK, and auxiliary peptides HBD-3 [84] and PADRE [49] were carefully selected based on their documented efficacy in enhancing the immune response. To aid detection and purification, we included six Histidine tags (HHHHH) at the carboxyl terminus of the MEBDB, which was named HP16118P.

### Prediction of the physicochemical and immunological properties of HP16118P

The ExPASy ProtParam server was used to predict the physicochemical properties of HP16118P, including molecular weight, theoretical isoelectric point, in vivo half-life, instability index, and overall average hydrophilicity following a previous study [85]. The Protein-Sol server was used to predict the solubility of the epitope molecules, with a value greater than 0.45 indicating easy solubility in water [86]. The IEDB Immunogenicity server was used to predict the immunogenicity of the epitope molecules, while VaxiJen v2.0 and ANTIGENpro servers were used to predict the antigenicity of HP16118P. AllerTOP v.2.0 and Allergen FP v.1.0 servers were employed to predict the allergenicity of HP16118P [78]. The ToxinPred server was used to predict the toxicity of HP16118P.

### Prediction of the spatial structure of HP16118P

In the case of HP16118P, understanding its structure can aid in elucidating its biological function and potential diagnostic applications. It can provide insights into its stability, interactions with its target molecules, and potential for interaction with other immune system components. This information can contribute to a better understanding of HP16118P's role as a biomarker for TB. Herein, the PSIPRED tool was used to predict the secondary structure of HP16118P, including the proportions of alpha-helices, beta-sheets, and random coils [87]. The I-TASSER (https://zhanggroup.org//I-TASSER/), Rebetta (https://robetta.bakerlab.org/), Swiss model (https://swissmodel.expasy.org/), and AlphaFold2 (https://colab.research.google.com/github/sokrypton/ColabFold/blob/main/AlphaFold2.ipynb) server was used to predict the three-dimensional (3D) structure of HP16118P. GalaxyWEB server (https://galaxy.seoklab.org/cgi-bin/submit.cgi?type=REFINE) was used to promote the 3D structure quality [88]. The quality of the constructed 3D model was further evaluated using the UCLA-DOE LAB - SAVES v6.0 server (https://saves.mbi.ucla.edu/). Specifically, the PROCHECK module was used to assess the overall quality of the constructed 3D structure and generate a Ramachandran plot [89]. The ERRAT module was employed to identify amino acid residues with correct and incorrect distributions in the protein structure [90], and the quality of all amino acid positions in the model was evaluated based on VERIFY 3D [91].

### Docking simulation of HP16118P with toll-like receptor 2

The ClusPro 2.0 online server (https://cluspro.bu.edu/home.php) was used to simulate the interaction between the MEBDB candidate and TLR-2 [92], and the hydrophobic interactions and hydrogen bonds were visualized using the LigPlot^+^ program [93]. The PDB file of TLR-2 (PDB ID: 6NIG) was obtained from the Molecular Modeling Database (MMDB) at the NCBI (https://www.ncbi.nlm.nih.gov/structure/).

### Simulation of the immune response induced by HP16118P

The C-ImmSim server (https://kraken.iac.rm.cnr.it/C-IMMSIM/) was used to predict the ability of HP16118P to induce immune cells to produce specific antibodies and various cytokines. This server can also assess the immune response of B lymphocyte populations and T lymphocyte populations [94].

### Cloning and purification of the expressed fusion protein HP16118P

The fusion protein HP16118P was synthesized by Shanghai Gene-Optimal Science & Technology Co., Ltd. The target gene of the epitope molecule HP16118P was inserted into the BamH I and Xho I restriction sites of the pET28a(+) plasmid. *Escherichia coli* (*E. coli*) was chosen as the host for cloning and expressing the fusion protein due to its common presence, fast reproduction, simple genome, and ease of manipulation. The protein solution was purified using Ni-affinity chromatography, and the quality of HP16118P was assessed using sodium dodecyl sulfate-polyacrylamide gel electrophoresis (SDS-PAGE). The purified PP16118P protein was subjected to endotoxin removal using the Beyotime Protein Endotoxin Removal Kit (Cat. No. C0268S, Beyotime, Shanghai, China). Subsequently, the endotoxin content in the PP16118P protein was determined using the Beyotime Chromogenic LAL Endotoxin Assay Kit (Cat. No. C0276S, Beyotime, Shanghai, China) following the manufacturer's instructions.

### Participant recruitment, inclusion and exclusion criteria, and medical ethics

This study recruited three groups of individuals, including HCs, LTBI, and ATB, from April to December 2022. The inclusion criteria for the HCs group were: no history of contact with ATB patients and negative IFN-γ assays, absence of clinical manifestations of TB, normal chest X-ray findings, exclusion of ATB diagnosis, and HIV-negative status. The exclusion criteria were: travel or residency in high-risk TB areas, employees of TB specialty hospitals or laboratories, children under 12 years old, individuals with a history of TB or old lung lesion on imaging, individuals unable to undergo CE (CFP-10/ESAT-6) antigen testing or allergies, HIV-positive individuals unable to undergo CE antigen testing or with allergies.

The inclusion criteria for the LTBI group were: close contact history with ATB patients or employees of TB specialty hospitals or laboratories, positive IFN-γ assays, absence of clinical manifestations of TB, normal chest X-ray findings, exclusion of ATB diagnosis, age 12 or older, and HIV-negative status. The exclusion criteria were diagnosed or suspected TB patients, pregnant or lactating women, individuals who have received more than one month of anti-TB treatment in the past, children under 12 years old, HIV-positive individuals unable to undergo CE antigen testing, or those with allergies.

The inclusion and exclusion criteria for ATB patients followed the "Tuberculosis Diagnostic Criteria (WS288-2017)" issued by China's National Health and Family Planning Commission. For detailed information on ATB patients' inclusion and exclusion criteria, please refer to our previous publication [34]. The research protocol and experiments were approved and supervised by the Ethics Committee of the Eighth Medical Center of the PLA General Hospital (Approval No: 309202204080808). This study was conducted following the Helsinki Declaration. Each participant agreed to participate in the study and disclose the laboratory data of their blood samples with informed consent.

### Differential analysis of IFN-γ^+^ T lymphocyte counts induced by HP16118P and 22 epitopes in the three groups of individuals using ELISPOT

The HCs (*n*=23), ATB patients (*n*=19), and LTBI individuals (*n*=24) were recruited in this study. Five milliliters of peripheral blood were collected from the three groups of individuals, and PBMCs were extracted. Subsequently, PBMCs were stimulated *in vitro* with AIM, CE (positive control), 12 HTL epitopes, 10 CTL epitopes, and HP16118P. The differential counts of IFN-γ^+^ T lymphocytes induced by HP16118P and 22 epitopes (refer to Table S6 for specific epitope sequences) were detected among the three groups of individuals using the human ELISPOT assay kit (Mabtech AB, Nacka Strand, Sweden).

### High-throughput liquid phase protein analysis to detect cytokine levels induced by HP16118P

To further elucidate the potential diagnostic value of HP16118P in ATB and LTBI, high-throughput liquid phase protein analysis was used to detect the levels of cytokines produced by PBMCs in the HCs, LTBI, and ATB groups induced by HP16118P. The experiment recruited 7 HC cases, 8 LTBI cases, and 7 ATB cases. Five milliliters of sterile venous blood anticoagulated with EDTA-2K were collected, and peripheral blood PBMCs were extracted. AIM (negative control), CE (CFP-10/ESAT-6) fusion protein (positive control), and HP16118P were added to the 96-well cell culture plate in a volume of 50 µl per well. A suspension of 100 µl PBMC cells was added to each well and cultured in a 37°C, 5% CO2 incubator for 48 hours. Then, the culture supernatant in each well was gently aspirated and transferred to 1.5 ml centrifuge tubes for further analysis. High-throughput liquid phase protein analysis was used to detect the levels of 35 cytokines induced by HP16118P in PBMCs of the HCs, LTBI, and ATB groups, including G-CSF, GM-CSF, HGF, IFN-α, IFN-γ, IL-1α, IL-1β, IL-10, IL-12p70, IL-13, IL-17F, IL-2, IL-21, IL-22, IL-23, IL-3, IL-31, IL-4, IL-5, IL-6, IL-8, IL-9, IP-10, MCP-1, MCP-3, MIG, MIP-1α, MIP-1β, PD-1, stromal cell-derived factor-1α (SDF-1α), TIM-3, TIMP-1, TNF-α, VEGF-A, and VEGF-R2. The potential of cytokines in distinguishing diagnosis among ATB, LTBI, and HCs was further analyzed using the receiver operator characteristic (ROC) curve.

### Machine learning algorithms to construct ATB and LTBI differential diagnostic models

The expression levels of 35 cytokines induced by HP16118P of participants in the ATB, LTBI, and HCs groups were statistically analyzed using the R package "autoReg". Specifically, subgroup analyses were conducted using ATB/LTBI/HC grouping as the primary observational indicator and PBS/HP16118P stimulation grouping as the secondary observational indicator. Normally-distributed variables were presented as mean ± SD, and differences between groups were analyzed using t-tests (*P*<0.05). Non-normally distributed variables were presented as median (IQR), and differences between groups were analyzed using Wilcoxon tests (*P* <0.05). Based on these analyses, a logistic regression model was constructed using the R package "glmnet", and univariate, multivariate, and stepwise logistic regression analyses were performed using the R package "autoReg". The variables selected in the stepwise logistic regression were considered qualified variables for machine learning modeling. The R package "mlr3" was utilized to construct 15 machine learning models. To provide a more comprehensive description of the diagnostic performance of the models from multiple perspectives, the diagnostic performance of the models was evaluated using 19 evaluation indicators based on the confusion matrix. The results from multiple models and indicators were visualized using the Chiplot online server (https://www.chiplot.online/) for heatmap visualization.

### Statistical analysis

All data in this study were analyzed and plotted using GraphPad Prism 10.0.0 software (San Diego, California, USA). For comparisons between two groups, a non-parametric t-test [data presented as mean with standard error of the mean (SEM)] or Mann-Whitney test (data presented as median with interquartile range) was used based on data normality. For experiments with three or more groups, one-way ANOVA (data presented as mean with SEM) or the Kruskal-Wallis test (data presented as median with interquartile range) was selected based on data normality and homogeneity of variance. A *P*-value < 0.05 indicates statistically significant differences. In principal component analysis, the method for selecting principal components (PCs) was based on eigenvalues greater than 1. The diagnostic sensitivity and specificity of HP16118P were analyzed using ROC curves, with an area under the curve (AUC) between 0.5 and 1 indicating good diagnostic model performance, and the closer the value is to 1, the better the performance. Pearson's correlation analysis was used to analyze the correlation between cytokines.

### Supplementary Information

**Supplementary Material 1.** 

## Data Availability

All the data supporting the findings of this study are available within the article and are available from the corresponding author upon request.

## Abbreviations

APCs: Antigen-presenting cells
ATB: Active tuberculosis
AIM: Auto induction medium
AUC: Area under the curve
ANOVA: One-way analysis of variance
BCG: Bacillus Calmette-Guérin
CFP-10: culture filtrate protein 10
CTL: Cytotoxic T lymphocyte
DCs: Dendritic cells
ESAT-6: Early secreted antigen target protein 6
ELISPOT: Enzyme-linked immunospot assays
EPs: Epithelial cells
G-CSF: Granulocyte-colony stimulating factor
GM-CSF: Granulocyte-macrophage colony-stimulating factor
HBD-3: Human beta-defensin-3
HCs: Healthy controls
HGF: Hepatocyte growth factor
HTL: Helper T lymphocyte
HIV: Human immunodeficiency virus
IEDB: Immune Epitope Database
IGRAs: Interferon-γ release assays
IFN-γ: Interferon-gamma
IL: Interleukin
IP-10: Interferon gamma inducible protein-10
LTBI: Latent tuberculosis infection
MA: Macrophages
MCP: Monocyte chemoattractant protein
MEBDBs: Multi-epitope-based diagnostic biomarkers
MEVs: Multi-epitope vaccines
MTB: *Mycobacterium tuberculosis*
MHC: Major histocompatibility complex
MIG: Monokine induced by gamma
MIP: Macrophage inflammatory protein
MMP: Matrix metalloproteinases
NCBI: National Centre for Biotechnological Information
NK cells: Natural killer cells
PBMCs: Peripheral blood mononuclear cells
PCs: Principal components
PD-1: Programmed cell death-1
PPD: Purified protein derivative tuberculin
PSMα4: Phenol-soluble modulin α 4
ROC: receiver operator characteristic
SDF-1α: Stromal cell-derived factor-1α
SDS-PAGE: Sodium dodecyl sulfate-polyacrylamide gel electrophoresis
SEM: Standard error of the mean
TB: Tuberculosis
TST: Tuberculin skin test
TLR-2: Toll-like receptor 2
TIM-3: T cell immunoglobulin and mucin domain-containing protein 3
TIMP-1: Tissue inhibitor of metalloprotease-1
TNF-α: Tumor necrosis factor α
Tregs: Regulatory T cells
TGF-β: Transforming growth factor-β
VEGF-A: Vascular endothelial growth factor A
VEGF-R2: Vascular endothelial growth factor receptor 2
WHO: World Health Organization
