# Supplementary material for: Construction of novel multi-epitope-based diagnostic biomarker HP16118P and its application in the differential diagnosis of Mycobacterium tuberculosis latent infection
Source: Mol Biomed. 2024 Apr 29;5:15. doi: 10.1186/s43556-024-00177-z (PMC11056354; doi:10.1186/s43556-024-00177-z)
Supplement: Supplementary file 1 — Supplementary Material 1. [file 43556_2024_177_MOESM1_ESM.docx]

**Construction of novel multi-epitope-based diagnostic biomarker HP16118P and its application in the differential diagnosis of *Mycobacterium tuberculosis* latent infection**

**Authors:** Jie Wang ^1,2#^, Fan Jiang ^1,3,5#^, Peng Cheng ^1^, Zhaoyang Ye ^1,4^, Linsheng Li ^1,4^, Ling Yang ^1,4^, Li Zhuang ^1,4^, Wenping Gong ^1, *^

^#^ These authors have contributed equally to this work.

**Running title:** A novel MEBDB for LTBI diagnosis.

**Affiliate:** ^1^ Beijing Key Laboratory of New Techniques of Tuberculosis Diagnosis and Treatment, Senior Department of Tuberculosis, The Eighth Medical Center of PLA General Hospital, Beijing 100091, China; ^2^ Department of Clinical Laboratory, The Eighth Medical Center of PLA General Hospital, Beijing 100091, China; ^3^ Section of Health, No. 94804 Unit of the Chinese People's Liberation Army, Shanghai, 200434, China; ^4^ Hebei North University, Zhangjiakou 075000, Hebei, China; ^5^ Resident standardization training cadet corps, Air Force Hospital of Eastern Theater, Nanjing, 210002, China.

**^*^Corresponding authors:** Wenping Gong (gwp891015@whu.edu.cn), The Eighth Medical Center of PLA General Hospital, 17^#^ Heishanhu Road, Haidian District, Beijing 100091, China.

**
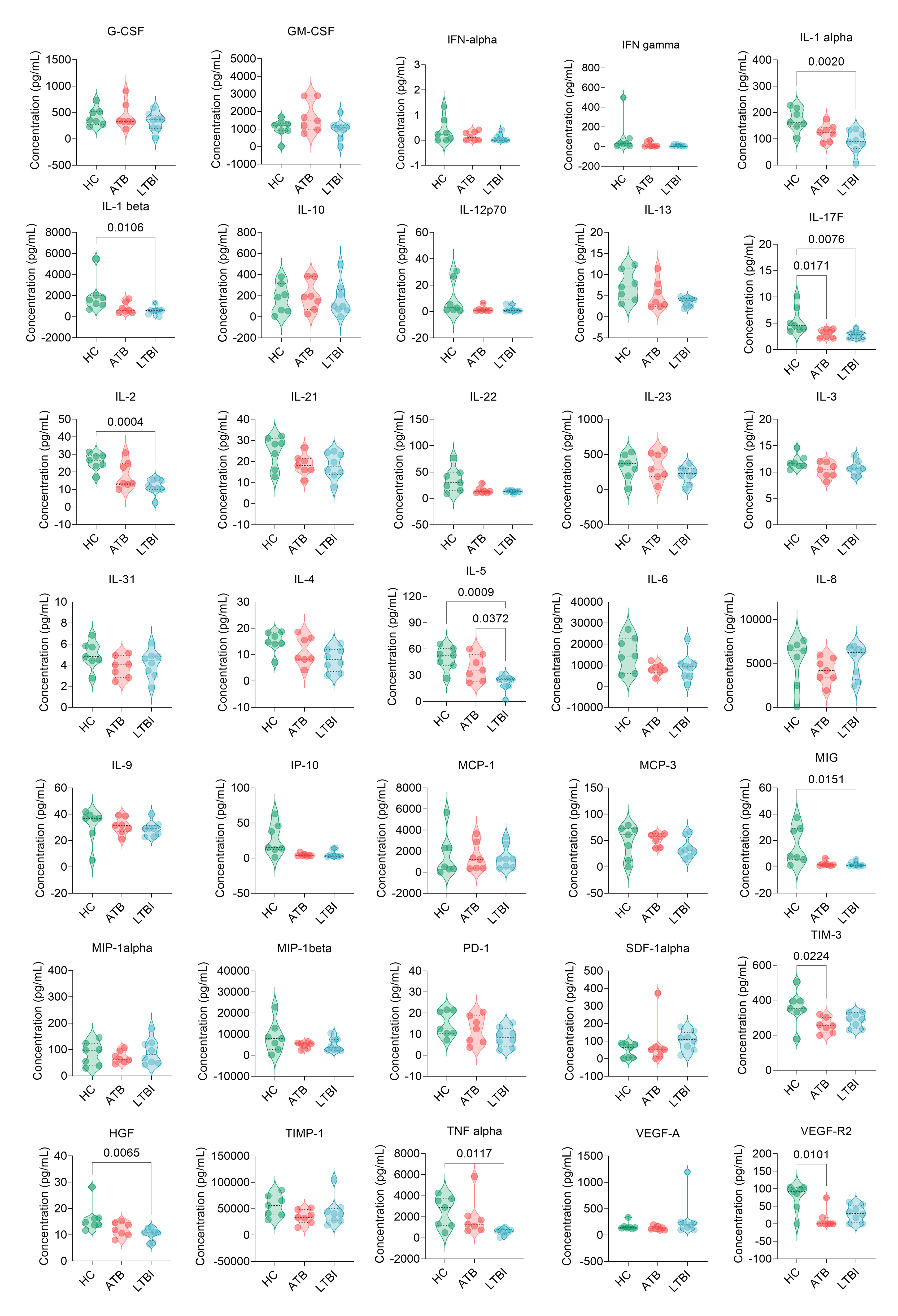
**

**Figure S1.** Comparison of the levels of 35 cytokines induced by HP16118P in the three groups.

**
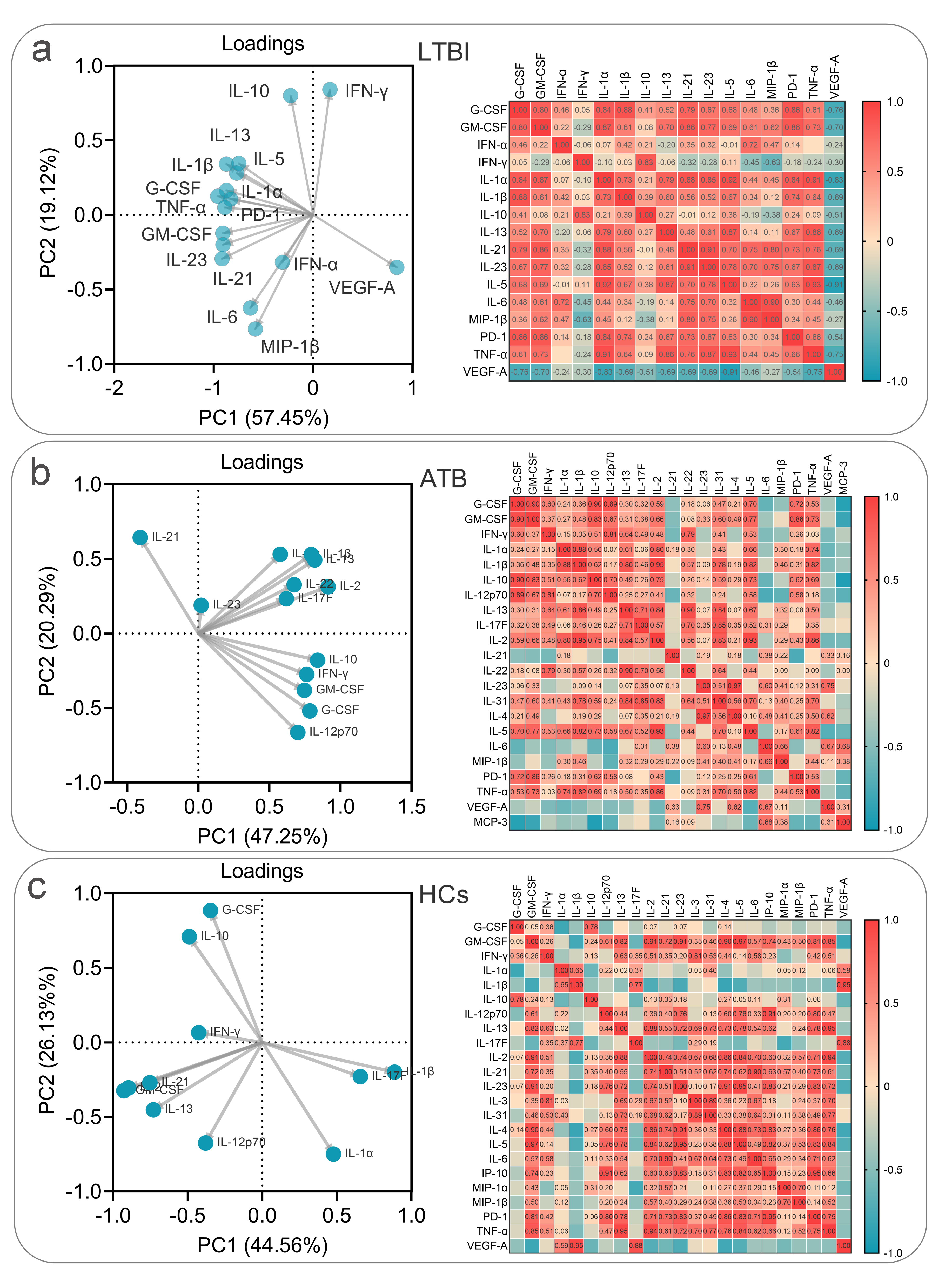
**

**Figure S2.** Principal component analysis (PCA) of HP16118P-induced cytokines in LTBI individuals, ATB patients, and healthy controls (HCs). Using GraphPad Prism 10.0.0 software, PCA and Pearson's R were used to analyze the potential relationships between 35 cytokines induced by HP16118P in the LTBI (A), ATB (B), and HCs (C) groups. Scatter plot showing the distribution of cytokines in three groups based on the major components PC1 and PC2. PC1 represents the primary patterns of variation, accounting for X% of the cumulative variance, while PC2 captures additional variation, accounting for Y% of the cumulative variance. The correlation circles represent the associations among specific cytokines, with positive correlations indicated by proximity and negative correlations by opposite directions. The distinct characteristics represented by PC1 and PC2, along with the corresponding cytokine correlation patterns, provide insights into the unique cytokine response profiles induced by HP16118P in different study cohorts. A *P*-value < 0.05 indicates a statistically significant difference.

**Table S1: List of information on the 15 LTBI-RD-associated antigens**

| Locus | Gene name | Product | antigen length | Function |
| --- | --- | --- | --- | --- |
| Rv1736c | *narX* | Probable nitrate reductase NarX | 652 | Mycobacterium tuberculosis expresses up-regulated proteins in hypoxia that induce the secretion of multiple cytokines by host peripheral blood immune cells. |
| Rv1737c | *narK2* | Possible nitrate/nitrite transporter NarK2 | 395 | (1) Higher IFN-γ^+^ TNF-α^+^ CD8^+^ T cells in LTBI vs. HC. (2) Higher TNF-α^+^ CD4^+^ T cells and IFN-γ^+^ TNF-α^+^ CD4^+^ T cells in LTBI vs. PTB. |
| Rv2626c | *hrp1* | Hypoxic response protein 1 Hrp1 | 143 | (1) higher IFN-γ producing T cells in CC vs. ATB and HC. (2) IFN-γ response to Rv2626c has shown positivity of 88.57% in CC and 7.5% in PTB. (3) 91% of CC QFT- subjects secreted low levels of IFN-γ, but 43% of HCWs QFT- people produced elevated IFN-γ, 69% of CC QFT+ subjects didn't produce IFN-γ to Rv2626c. (4) Sensitivity and specificity of Rv2626c in ATB was of 77.1% and 85.1% |
| Rv2656c | *Rv2656c* | Possible PhiRv2 prophage protein | 130 | Unknown |
| Rv2659c | *Rv2659c* | Probable PhiRv2 prophage integrase | 375 | higher IFN-γ producing T cells in LTBI vs. ATB and HC |
| Rv1511 | *gmdA* | GDP-D-mannose dehydratase GmdA | 340 | Unknown |
| Rv1980c | *mpt64* | Immunogenic protein Mpt64 | 228 | Sensitivity and specificity were 0.92 and 0.95 respectively, sensitivity of the MPT64 test was significantly higher in TB infected children than in adults. |
| Rv1981c | *nrdF1* | Ribonucleoside-diphosphate reductase (beta chain) NrdF1 | 322 | ELISPOT of Rv1981c achieved sensitivities of 60% in ATB and specificities of 90% in BCG-vaccinated HC. |
| Rv3873 | *PPE68* | PPE family protein PPE68 | 368 | Sensitivity and specificity of PE68 for detecting LTBI in children were 73% and 75%. |
| Rv3878 | *espJ* | ESX-1 secretion-associated protein EspJ | 280 | Elicited stronger immunoreactivity and could discriminate TB from HC vaccinated with BCG. |
| Rv3879c | *espK* | ESX-1 secretion-associated protein EspK | 729 | The immunodominance of Rv3879c is higher than that of Rv3878 and Rv3873 in ATB and LTBI subjects. |
| Rv3425 | *PPE57* | PPE family protein PPE57 | 176 | (1) Rv0310c-E coupled with Rv3425, sensitivity: 87.30%, specificity: 73.68% had the strongest performance for diagnostics of ATB. (2) Rv3425 have the promising potential to distinguish ATB from HC vaccinated with BCG. |
| Rv3429 | *PPE59* | PPE family protein PPE59 | 178 | ELISPOT of Rv3429 achieved sensitivities of 47% in ATB and specificities of 93% in BCG-vaccinated HC. |
| Rv1978 | *Rv1978* | Conserved protein | 282 | ELISPOT of Rv1978 achieved sensitivities of 59% in ATB and specificities of 94% in BCG-vaccinated HC |
| Rv2031c | *hspX* | Heat shock protein HspX | 144 | (1) Higher concentrations of IFN-γ in LTBI vs. ATB vs. HC. (2) Higher IFN-γ^+^ TNF-α^+^ CD8^+^ T cells in LTBI vs. HCs. (3) Lower IFN-γ, IL-10, TNF-α in LTBI vs. ATB & HC |

**Table S2. Physicochemical and immunological properties of HP16118P.**

| Parameters | | Servers | Results |
| --- | --- | --- | --- |
| Physicochemical properties | Molecular weight (MW) | Expasy ProtParam | 90265.44 |
|  | Isoelectric point (pl) | Expasy ProtParam | 9.84 |
|  | Instability index | Expasy ProtParam | 43.02 |
|  | Aliphatic index | Expasy ProtParam | 75.09 |
|  | Grand average of hydropathicity (GRAVY) | Expasy ProtParam | -2.7 |
|  | Estimated half-life | Expasy ProtParam | 30 hours (mammalian reticulocytes, in vitro).  >20 hours (yeast, in vivo).  >10 hours (Escherichia coli, in vivo). |
|  | Isoelectric point (pl) | Protein–Sol | 10.23 |
|  | Solubility | Protein–Sol | 0.382 |
| Immunological properties | Antigenicity | Vaxijen 2.0 | 0.7381 |
|  |  | ANTIGEN pro | 0.60063 |
|  | Allergenicity | Aller TOP | non-allergen |
|  |  | Aller FP | non-allergen |
|  | Immunogenicity | IEDB | 6.43254 |
|  | Toxicity | Toxin Pred | non-toxin |

**Table S3. Sensitivity and specificity of IL-5 and IL-17F cytokines induced by the HP16118P in the diagnosis of ATB and LTBI.**

| Cytokines | Groups | *P* value | AUC | 95% CI | Cutoff value (pg/mL) | Sensitivity | Specificity |
| --- | --- | --- | --- | --- | --- | --- | --- |
| IL-5 | ATB *vs.* HC | 0.1599 | 0.7245 | 0.4498 to 0.9991 | - | - | - |
|  | ATB *vs.* LTBI | **0.0372** | 0.8214 | 0.5843 to 1.000 | < 31.36 | 100% | 71.43% |
|  | HC *vs.* LTBI | **0.0026** | 0.9643 | 0.8770 to 1.000 | < 0.0900 | 100% | 85.71% |
| IL-17F | ATB *vs.* HC | **0.0088** | 0.9184 | 0.7716 to 1.000 | < 3.645 | 71.43% | 85.71% |
|  | ATB *vs.* LTBI | 0.2976 | 0.6607 | 0.3664 to 0.9551 | - | - | - |
|  | HC *vs.* LTBI | **0.0038** | 0.9464 | 0.8299 to 1.000 | < 3.645 | 87.50% | 85.71% |
| IL-5+IL-17F | ATB *vs.* HC | 0.1478 | 0.6607 | 0.4544 to 0.8670 | - | - | - |
|  | ATB *vs.* LTBI | 0.1575 | 0.6518 | 0.4507 to 0.8529 | - | - | - |
|  | HC *vs.* LTBI | **0.0159** | 0.7589 | 0.5842 to 0.9336 | < 3.900 | 50% | 85.71% |

**Table S4. Results of single-factor, multi-factor, and stepwise logistic regression analysis for 35 cytokines.**

| Cytokines | desc | ATB (N=7) | LTBI (N=8) | OR (univariable) | OR (multivariable) | OR (final) |
| --- | --- | --- | --- | --- | --- | --- |
| GM_CSF | Mean ± SD | 1686.9 ± 876.7 | 986.8 ± 571.0 | 1.00 (1.00-1.00, p=.131) | 1.09 (0.00-Inf, p=1.000) | 1.16 (0.00-5.5918E+140, p=.999) |
| IL_23 | Mean ± SD | 332.4 ± 198.7 | 211.6 ± 122.6 | 0.99 (0.99-1.00, p=.177) | 0.96 (0.00-Inf, p=1.000) | 0.72 (0.00-Inf, p=.999) |
| IL_5 | Mean ± SD | 38.6 ± 14.8 | 21.3 ± 9.1 | 0.84 (0.68-1.03, p=.098) | 0.00 (0.00-Inf, p=1.000) | 0.00 (0.00-Inf, p=.999) |
| MCP_3 | Mean ± SD | 52.3 ± 11.9 | 36.2 ± 18.8 | 0.94 (0.87-1.01, p=.086) | 0.03 (0.00-Inf, p=1.000) | 0.01 (0.00-Inf, p=.999) |
| IL_1_alpha | Mean ± SD | 124.0 ± 31.8 | 88.9 ± 43.8 | 0.97 (0.93-1.01, p=.130) | 0.52 (0.00-Inf, p=1.000) |  |
| HGF | Mean ± SD | 12.0 ± 2.7 | 10.2 ± 2.3 | 0.72 (0.44-1.18, p=.190) | 1.5053E+37 (0.00-Inf, p=1.000) |  |
| IL_2 | Mean ± SD | 18.4 ± 7.8 | 11.6 ± 4.5 | 0.80 (0.60-1.06, p=.116) | 1.9023E+14 (0.00-Inf, p=1.000) |  |
| TIM_3 | Mean ± SD | 256.0 ± 43.6 | 286.8 ± 41.9 | 1.02 (0.99-1.05, p=.181) | 0.11 (0.00-Inf, p=1.000) |  |
| TNF_alpha | Mean ± SD | 1925.2 ± 1791.9 | 590.7 ± 305.9 | 0.99 (0.99-1.00, p=.088) | 0.98 (0.00-Inf, p=1.000) |  |
| VEGF_A | Mean ± SD | 131.3 ± 38.6 | 306.3 ± 365.5 | 1.02 (0.99-1.04, p=.124) | 0.68 (0.00-Inf, p=1.000) |  |
| G_CSF | Mean ± SD | 438.0 ± 248.8 | 322.9 ± 177.9 | 1.00 (0.99-1.00, p=.308) |  |  |
| IFN_alpha | Mean ± SD | 0.2 ± 0.2 | 0.1 ± 0.2 | 0.07 (0.00-47.32, p=.417) |  |  |
| IFN_gamma | Mean ± SD | 20.0 ± 27.6 | 9.8 ± 9.5 | 0.97 (0.91-1.03, p=.340) |  |  |
| IL_1_beta | Mean ± SD | 822.0 ± 529.7 | 566.8 ± 367.8 | 1.00 (1.00-1.00, p=.283) |  |  |
| IL_10 | Mean ± SD | 199.4 ± 140.1 | 166.4 ± 159.1 | 1.00 (0.99-1.01, p=.653) |  |  |
| IL_12p70 | Mean ± SD | 1.8 ± 2.1 | 1.8 ± 2.3 | 1.01 (0.62-1.67, p=.955) |  |  |
| IL_13 | Mean ± SD | 5.2 ± 3.4 | 3.6 ± 1.0 | 0.71 (0.39-1.27, p=.247) |  |  |
| IL_17F | Mean ± SD | 3.1 ± 0.8 | 2.8 ± 0.7 | 0.64 (0.15-2.79, p=.557) |  |  |
| IL_21 | Mean ± SD | 18.5 ± 5.0 | 18.1 ± 6.0 | 0.98 (0.81-1.20, p=.875) |  |  |
| IL_22 | Mean ± SD | 15.3 ± 6.5 | 13.3 ± 2.5 | 0.90 (0.69-1.17, p=.429) |  |  |
| IL_3 | Mean ± SD | 10.4 ± 1.4 | 10.8 ± 1.3 | 1.28 (0.57-2.87, p=.543) |  |  |
| IL_31 | Mean ± SD | 3.8 ± 1.0 | 4.1 ± 1.3 | 1.26 (0.50-3.21, p=.622) |  |  |
| IL_4 | Mean ± SD | 11.4 ± 5.3 | 8.1 ± 4.4 | 0.86 (0.67-1.09, p=.201) |  |  |
| IL_6 | Mean ± SD | 8106.7 ± 2618.2 | 9198.0 ± 6471.1 | 1.00 (1.00-1.00, p=.661) |  |  |
| IL_8 | Mean ± SD | 4207.9 ± 1428.5 | 5286.0 ± 1856.7 | 1.00 (1.00-1.00, p=.220) |  |  |
| IL_9 | Mean ± SD | 31.2 ± 6.4 | 28.8 ± 5.6 | 0.93 (0.77-1.12, p=.427) |  |  |
| IP_10 | Mean ± SD | 4.5 ± 2.0 | 5.7 ± 5.1 | 1.09 (0.82-1.46, p=.555) |  |  |
| MCP_1 | Mean ± SD | 1466.8 ± 1321.5 | 1399.9 ± 1080.3 | 1.00 (1.00-1.00, p=.908) |  |  |
| MIG | Mean ± SD | 2.2 ± 2.0 | 1.9 ± 1.8 | 0.93 (0.52-1.67, p=.812) |  |  |
| MIP_1alpha | Mean ± SD | 71.2 ± 22.7 | 91.6 ± 48.3 | 1.02 (0.98-1.05, p=.315) |  |  |
| MIP_1beta | Mean ± SD | 4799.9 ± 1483.0 | 4912.3 ± 3114.6 | 1.00 (1.00-1.00, p=.926) |  |  |
| PD_1 | Mean ± SD | 12.1 ± 6.5 | 8.5 ± 4.6 | 0.88 (0.72-1.08, p=.231) |  |  |
| SDF_1alpha | Mean ± SD | 87.0 ± 128.4 | 102.8 ± 54.3 | 1.00 (0.99-1.01, p=.736) |  |  |
| TIMP_1 | Mean ± SD | 34651.7 ± 13061.7 | 44545.1 ± 26870.7 | 1.00 (1.00-1.00, p=.392) |  |  |
| VEGF_R2 | Mean ± SD | 13.1 ± 27.8 | 29.4 ± 23.1 | 1.03 (0.98-1.08, p=.229) |  |  |

**Table S5. Detailed results of machine learning modeling and evaluation.**

| learner id | class if.ce | Accuracy | Kappa | Accuracy Lower | Accuracy Upper | Accuracy Null | Accuracy P Value | Mcnemar P Value | Sensitivity | Specificity | Pos Pred Value | Neg Pred Value | Precision | Recall | F1 | Prevalence | Detection Rate | Detection Prevalence | Balanced Accuracy |
| --- | --- | --- | --- | --- | --- | --- | --- | --- | --- | --- | --- | --- | --- | --- | --- | --- | --- | --- | --- |
| classif.cv_glmnet | 0.5333 | 0.8667 | 0.7273 | 0.5954 | 0.9834 | 0.5333 | 0.0076 | 0.4795 | 1.0000 | 0.7143 | 0.8000 | 1.0000 | 0.8000 | 1.0000 | 0.8889 | 0.5333 | 0.5333 | 0.6667 | 0.8571 |
| classif.debug | 0.4000 | 0.5333 | 0.0000 | 0.2659 | 0.7873 | 0.5333 | 0.6037 | 0.0233 | 1.0000 | 0.0000 | 0.5333 | 0.0000 | 0.5333 | 1.0000 | 0.6957 | 0.5333 | 0.5333 | 1.0000 | 0.5000 |
| classif.featureless | 0.6667 | 0.5333 | 0.0000 | 0.2659 | 0.7873 | 0.5333 | 0.6037 | 0.0233 | 1.0000 | 0.0000 | 0.5333 | 0.0000 | 0.5333 | 1.0000 | 0.6957 | 0.5333 | 0.5333 | 1.0000 | 0.5000 |
| classif.glmnet | 0.2667 | 0.8667 | 0.7321 | 0.5954 | 0.9834 | 0.5333 | 0.0076 | 1.0000 | 0.8750 | 0.8571 | 0.8750 | 0.8571 | 0.8750 | 0.8750 | 0.8750 | 0.5333 | 0.4667 | 0.5333 | 0.8661 |
| classif.kknn | 0.2000 | 0.8667 | 0.7321 | 0.5954 | 0.9834 | 0.5333 | 0.0076 | 1.0000 | 0.8750 | 0.8571 | 0.8750 | 0.8571 | 0.8750 | 0.8750 | 0.8750 | 0.5333 | 0.4667 | 0.5333 | 0.8661 |
| classif.lda | 0.2667 | 0.8667 | 0.7321 | 0.5954 | 0.9834 | 0.5333 | 0.0076 | 1.0000 | 0.8750 | 0.8571 | 0.8750 | 0.8571 | 0.8750 | 0.8750 | 0.8750 | 0.5333 | 0.4667 | 0.5333 | 0.8661 |
| classif.log_reg | 0.2000 | 1.0000 | 1.0000 | 0.7820 | 1.0000 | 0.5333 | 0.0001 | 0.0000 | 1.0000 | 1.0000 | 1.0000 | 1.0000 | 1.0000 | 1.0000 | 1.0000 | 0.5333 | 0.5333 | 0.5333 | 1.0000 |
| classif.multinom | 0.2667 | 1.0000 | 1.0000 | 0.7820 | 1.0000 | 0.5333 | 0.0001 | 0.0000 | 1.0000 | 1.0000 | 1.0000 | 1.0000 | 1.0000 | 1.0000 | 1.0000 | 0.5333 | 0.5333 | 0.5333 | 1.0000 |
| classif.naive_bayes | 0.3333 | 0.8667 | 0.7321 | 0.5954 | 0.9834 | 0.5333 | 0.0076 | 1.0000 | 0.8750 | 0.8571 | 0.8750 | 0.8571 | 0.8750 | 0.8750 | 0.8750 | 0.5333 | 0.4667 | 0.5333 | 0.8661 |
| classif.nnet | 0.6667 | 0.4000 | -0.2162 | 0.1634 | 0.6771 | 0.5333 | 0.9022 | 1.0000 | 0.5000 | 0.2857 | 0.4444 | 0.3333 | 0.4444 | 0.5000 | 0.4706 | 0.5333 | 0.2667 | 0.6000 | 0.3929 |
| classif.qda | 0.2000 | 0.9333 | 0.8649 | 0.6805 | 0.9983 | 0.5333 | 0.0011 | 1.0000 | 1.0000 | 0.8571 | 0.8889 | 1.0000 | 0.8889 | 1.0000 | 0.9412 | 0.5333 | 0.5333 | 0.6000 | 0.9286 |
| classif.ranger | 0.2667 | 1.0000 | 1.0000 | 0.7820 | 1.0000 | 0.5333 | 0.0001 | 0.0000 | 1.0000 | 1.0000 | 1.0000 | 1.0000 | 1.0000 | 1.0000 | 1.0000 | 0.5333 | 0.5333 | 0.5333 | 1.0000 |
| classif.rpart | 0.7333 | 0.5333 | 0.0000 | 0.2659 | 0.7873 | 0.5333 | 0.6037 | 0.0233 | 1.0000 | 0.0000 | 0.5333 | 0.0000 | 0.5333 | 1.0000 | 0.6957 | 0.5333 | 0.5333 | 1.0000 | 0.5000 |
| classif.svm | 0.4667 | 0.8667 | 0.7321 | 0.5954 | 0.9834 | 0.5333 | 0.0076 | 1.0000 | 0.8750 | 0.8571 | 0.8750 | 0.8571 | 0.8750 | 0.8750 | 0.8750 | 0.5333 | 0.4667 | 0.5333 | 0.8661 |
| classif.xgboost | 0.3333 | 0.8667 | 0.7273 | 0.5954 | 0.9834 | 0.5333 | 0.0076 | 0.4795 | 1.0000 | 0.7143 | 0.8000 | 1.0000 | 0.8000 | 1.0000 | 0.8889 | 0.5333 | 0.5333 | 0.6667 | 0.8571 |

**Table S6. The list of peptides used in ELISPOT.**

| **Peptide No.** | **Peptide sequence** |
| --- | --- |
| HTL-3 | DFLPVVLEFAATVDPEA |
| HTL-5 | PAYNINISLPSYYPDQ |
| HTL-6 | DPAYNINISLPSYYPDQ |
| HTL-7 | SDPAYNINISLPSYYPDQ |
| HTL-13 | DLRVHDLRHSGAVLAAST |
| HTL-16 | VAPSVMPAAAAGSSAT |
| HTL-17 | GLSAAAAKLAGLVF |
| HTL-18 | DTGAGARPAASPLAAPV |
| HTL-19 | AASGVPGARAAAAAPS |
| HTL-20 | VTPAAASGVPGARAAAAA |
| HTL-21 | ATGREAAHLRAFRAYAAH |
| HTL-22 | TGREAAHLRAFRAYAAHS |
| CTL-1 | RGKGGLVRV |
| CTL-4 | ILPVLAVGV |
| CTL-5 | EMKEGRYEVR |
| CTL-6 | RRDAYIRRV |
| CTL-7 | VTDRTVRQM |
| CTL-8 | GIKDRTRAHY |
| CTL-9 | LRPTEVDSL |
| CTL-10 | VSIAPNAGL |
| CTL-11 | QAVELTARL |
| CTL-12 | EAAHLRAFR |
